# VOC·BIO Library: An Integrated Platform for Published Aphid GC-MS Evidence, Biological Contexts, Cheminformatics and Exploratory OBP Docking

**DOI:** 10.3390/insects17101062

**Published:** 2026-10-15

**Authors:** Taghreed Alsufyani, Taufia Hussain, Noura J. Alotaibi

**Affiliations:** 1 Department of Chemistry, College of Science, Taif University, P.O. Box 11099, Taif 21944, Saudi Arabia; 2 Department of Biosciences, University of Rostock, 18059 Rostock, Germany; 3 Department of Biology, College of Science, Taif University, P.O. Box 11099, Taif 21944, Saudi Arabia; n.alotaibi@tu.edu.sa

**Keywords:** volatile organic compounds, aphid chemical ecology, cheminformatics, GC-MS, insect–plant interactions, odorant-binding proteins, molecular docking, AutoDock Vina 1.2.7, structural similarity, semiochemicals, Saudi Arabia

## Abstract

Published aphid volatile records are difficult to explore when chemical annotations, identification confidence and biological context are stored separately. We developed VOC·BIO Library to integrate these evidence layers with reproducible computational tools. Version 1.0 preserves 81 published GC-MS Bio features across 15 biological and sample contexts, with 352 reported feature-context presence records. The features comprise 11 authentic-standard-supported annotations, 59 tentative library identifications and 11 unknowns. Seventy named candidate interpretations are linked to 55 curated molecular structures. Eleven interface features provide an overview dashboard, a published-feature catalogue, chemical structures and descriptors, biological contexts, descriptive statistics, evidence tracing, docking predictions, a feature-context network, structural similarity, source references and deterministic guided questions. The 81 × 15 presence matrix supports context prevalence, Jaccard similarity and average-linkage clustering. Twenty-eight features are listed in only one context. The full evidence network contains 190 nodes and 452 documented links. These are descriptive publication-based relationships, not biological replicate measurements or ecological effects. Structure-enabled comparisons use Morgan fingerprints and Tanimoto similarity without upgrading the published identification confidence. An additional exploratory study screens 20 candidates against MvicOBP3 and NribOBP3 in 200 docking runs with 2000 retained poses. Independent redocking controls distinguish pose recovery from score ranking. The platform therefore connects source evidence, biological context and complementary analyses within a traceable research environment. Docking, structural similarity and documented occurrence do not establish compound origin, binding affinity, behaviour or pest-control efficacy.

## Simple Summary

Aphids damage crops and interact with their host plants through chemical signals. Published volatile profiles contain useful information, but their interpretation depends on sample context and confidence in compound identification. VOC·BIO Library brings these records into one research environment. Its 11 features support data browsing, sample-context exploration, chemical structures, descriptive statistics, evidence tracing, structural similarity, networks, source references, guided questions and exploratory protein docking. Version 1.0 preserves 81 published GC-MS features across 15 contexts. It links 70 named candidate interpretations to 55 curated molecular structures without treating tentative identifications as confirmed compounds. The platform lets researchers follow a feature from its published annotation to its context, structure and computational outputs. A separate docking study evaluates 20 candidates against two aphid odorant-binding proteins. These predictions support future molecular studies and do not establish biological activity. The library is a defined, traceable starting resource for aphid chemical ecology, with broader datasets and experimental validation left for future development.

## 1. Introduction

Aphids (Hemiptera: Aphididae) are important agricultural pests. They damage plants through phloem feeding, honeydew production and virus transmission [1,2]. Species identification can be difficult because of their taxonomic diversity and variable morphology [3,4]. DNA barcoding helps resolve species identity. Chemical profiles can provide complementary information about the sampled material and its biological context. Volatile organic compounds (VOCs) produced by aphids and their host plants can participate in insect–plant and plant–natural-enemy interactions. Among these signals are (E)-beta-farnesene, an important aphid alarm pheromone [5], and alpha-pinene, which has also been reported as an alarm-pheromone component in some aphids [6]. Plant volatiles released during aphid feeding can attract parasitoids [7], and changes in host-associated emissions, including methyl salicylate and sesquiterpenes, have been associated with aphid infestation [8,9]. Insect olfaction involves protein classes with different roles. Soluble odorant-binding proteins (OBPs) can bind hydrophobic molecules in the sensillar environment, while membrane odorant receptors (ORs) contribute to neuronal detection [10,11,12]. These proteins should not be treated interchangeably. Structure-based OBP calculations provide one complementary view of candidate molecules. They require separate validation and cannot alone demonstrate physiological binding, receptor activation or behaviour.

Alotaibi et al. [13] demonstrated the use of aphid headspace profiles as chemical fingerprints by combining closed-loop stripping, GC-MS and canonical analysis of principal coordinates. The published supplementary dataset contains 81 Bio features across 15 sample/host location contexts representing 10 aphid species. Importantly, those features carry different identification-confidence categories, ranging from compounds verified with authentic standards to tentative library-based annotations and unknowns. A chromatographic feature, a proposed chemical identity and a molecular structure suitable for computational analysis therefore represent different levels of evidence. The same research group has subsequently examined VOC-mediated interactions in pomegranate–*Aphis punicae* [14] and grapevine–*Aphis illinoisensis* systems [15], as well as bioactive secondary metabolites associated with aphid endosymbiotic bacteria [16]. These studies motivate computational infrastructure that can preserve experimental provenance while connecting VOC evidence to chemical structures, biological context and complementary analyses.

Several established resources support related areas of chemical-ecology and mass-spectrometry research. PheroBase [17] provides information on pheromones and other semiochemicals across diverse organisms; mVOC 2.0 [18] focuses on microbial volatiles; GNPS [19] provides infrastructure for mass-spectrometry analysis and molecular networking; and MACE [20] provides curated electron-ionization mass spectra for chemical-ecology applications. These resources were developed for different purposes and provide capabilities complementary to VOC·BIO Library. The aim of VOC·BIO Library is not to replace them, but to provide a provenance-aware framework in which experimental aphid VOC evidence can be followed from reported GC-MS features and confidence levels to chemical interpretation, molecular structure, biological context and subsequent computational analysis.

A useful VOC platform should support more than a list of chemical names. Researchers need to inspect which sample contexts contain a reported feature, compare documentary occurrence patterns, examine candidate structures and follow each interpretation back to its source. These tasks use different observational units and different calculations. A feature-context similarity describes shared reporting. Molecular similarity describes a chosen structure representation. Neither directly establishes a shared biological effect. A common evidence model can connect these views while retaining their distinct meanings.

VOC·BIO Library addresses these needs through 11 connected interface features. They cover an overview dashboard, published features, chemical structures, biological and sample contexts, descriptive statistics, an Evidence Explorer, docking predictions, a feature-context network, structural similarity, references and a Research Team Assistant. The initial dataset is regional and publication-based. Its 81 GC-MS features and 15 contexts provide a bounded case study for the integration of evidence and computational methods. Seventy features have named chemical interpretations, and 55 have curated candidate structures. Unknowns and unresolved identities remain accessible as evidence records.

We hypothesized that separating published features, biological contexts and candidate structures through stable identifiers would allow the same evidence to be followed consistently across complementary analytical views without upgrading identification confidence. We examined this data-integration hypothesis through count reconciliation, identifier joins, reconstruction of the presence matrix and checks of the displayed evidence. The objectives were to preserve the published annotations and source confidence; implement and describe all 11 platform features; calculate reproducible context, structure and network summaries; and provide a separately benchmarked exploratory docking workflow for a defined candidate panel. This is a hypothesis about evidence integration. Experimental binding, behavioural activity and ecological function were not tested.

## 2. Materials and Methods

### 2.1. Source Dataset, Evidence Reconstruction and Provenance

The experimental foundation of VOC·BIO Library is the aphid headspace study of Alotaibi et al. [13] and its Supplementary Tables S1 and S2. Source Table S1 supplies biological and sample metadata. Source Table S2 supplies 81 Bio features across 15 contexts. We retained the identification-confidence categories reported in that study: 11 authentic-standard-supported features, 32 higher-confidence tentative library identifications, 27 lower-confidence tentative library identifications and 11 unknown features. The original notation was *, ?, ?? and UK, respectively. The reported NIST reverse-match ranges were 990–850 for the higher tentative category, 800–700 for the lower tentative category and below 700 for unknowns. These are inherited source categories, not new identity assessments by VOC·BIO. No new sampling or GC-MS instrument measurements were performed in the present computational study.

The platform separates the published GC-MS feature, its named candidate interpretation and its molecular structure. Seventy of the 81 features have named publication-derived annotations. Chemical curation supplied molecular representations for 55 candidates: 11 Tier A, 30 Tier B and 14 Tier C records. These tier labels track the corresponding source-confidence groups. Fifteen named interpretations remained unresolved or structurally ambiguous. The 11 unknown features received no inferred structures. Structure availability did not upgrade experimental identification confidence. Supplementary Tables S1, S10 and S11 and their complete CSV files retain the feature, context and chemical layers.

The source study reports 63 biological observations and three technical replicates per observation [13]. The surviving intermediate matrices do not reproduce the exact published 63 × 81 analysis. They include 75 × 84 and 76 × 84 historical stages, while a surviving historical analysis output contains 70 observations. The exact exclusions and crosswalk remain unresolved. We therefore use the published Table S2 presence records for platform summaries. We do not infer replicate means, quantitative abundance, peak origin or missing blank-control information. Supplementary Methods S4 records the historical normalization, annotation and matrix findings.

The source audit also identified duplicated or incomplete historical taxon labels and an unresolved Alhada rose assignment. The nine literal application labels represented seven nominal taxa after documentary spelling normalization. They were not a defensible species-curation result. The platform now preserves the 10 source-publication taxon labels and their 15 contexts. Conflicting documentary assignments remain visible. Supplementary Methods S4 and Table S10 explain the labels and the foxtail/feed locality discrepancies.

A historical import produced counts of 767 processed records, 429 records marked as identified, 17 imported names and 117 context associations. The audit found a global compound-label import defect and separate spreadsheet layout shifts. Those counts cannot establish a validated chemical inclusion sequence. The scientific layer described here is anchored directly to published Table S2. Historical parser outputs and unverified behavioural fields are kept outside the publication-facing evidence layer.

### 2.2. Evidence Architecture Dashboard and Feature Catalogue

**Figure 1:**
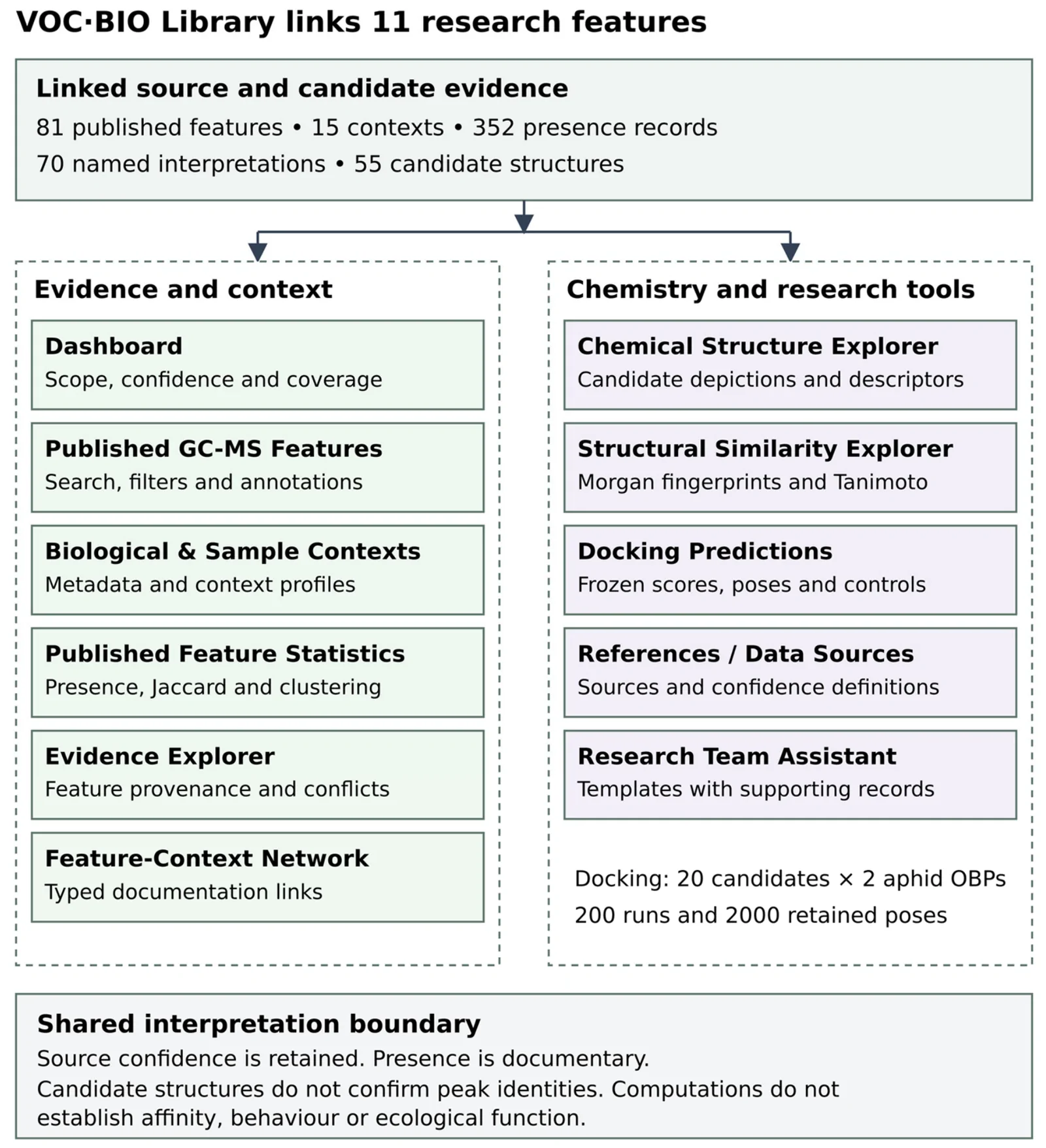
The 11 research features of VOC·BIO Library and their shared evidence layers. Each view retains the distinction between published presence, candidate interpretation and molecular structure. Docking reads finalized offline results for a defined subset. No feature upgrades source confidence or establishes biological activity from a computation.

VOC·BIO Library uses linked, read-only evidence tables and a Streamlit interface (Figure 1). Features, contexts, presence assertions, candidate chemical entities, feature–chemical mappings and provenance records have separate identifiers. One row in the feature catalogue represents one published Bio feature. A presence row represents one reported feature-context association. Query services join these records with explicit one-to-one or many-to-one checks. Context filtering selects linked features and then returns unique feature rows. This prevents repeated detections from being counted as new chemicals.

The Dashboard computes the number of features, contexts, presence records, named interpretations, structure-enabled candidates and unknowns in the selected view. It also displays confidence composition and the most widely reported features. Published GC-MS Features provides text search, confidence, species, host, environment, structure-status and prevalence filters. Bio identifiers are ordered by their numerical component, ensuring that, for example, Bio2 precedes Bio10. Retention times and published annotations remain unchanged source fields. Filtered tables can be exported as CSV files. Main Table 1 summarizes the 11 features and their computational roles.

### 2.3. Biological and Sample Contexts

Biological and Sample Contexts joins published presence records to source sample metadata. The catalogue retains common sample name, aphid taxon, accession, host plant and part, locality, coordinates, date and documented collection environment. Filters operate on species, host, plant part, environment and literal text. Species-host profiles aggregate unique contexts and linked features. They are documentary summaries and do not estimate biological replicate prevalence. Historical sample-associated masses are displayed separately because the weighed material and their analytical propagation remain unresolved. No mass is silently joined to a published presence value.

### 2.4. Chemical Structures and Molecular Descriptors

Chemical Structure Explorer operates on the 55 publication-linked candidate structures. The interface connects the selected feature to candidate name, PubChem CID, formula, SMILES, InChIKey, identification confidence and curation status. Candidate identity and experimental peak identity are displayed separately. Molecular depictions use PubChem image requests [21] from a CID or an encoded SMILES. Descriptor calculation is local and uses RDKit 2026.03.1 [22]. External depiction requests do not validate a chromatographic assignment.

The computed properties are exact molecular mass, Wildman–Crippen LogP, topological polar surface area (TPSA), hydrogen-bond donors and acceptors, rotatable bonds, total and aromatic ring counts, heavy-atom count and fraction of sp3 carbon. The interface labels exact mass as molecular weight. A radar display divides five descriptors by fixed display scales and clips the plotted values to the interval from zero to one. The numerical table retains the calculated values. The plot is a navigation aid, not a calibrated volatility or bioactivity model. The former mass/LogP/TPSA volatility heuristic is not used to support scientific conclusions.

### 2.5. Published Feature Statistics

Published Feature Statistics builds an 81 × 15 binary matrix from unique feature-context presence assertions. A value of one means reported present. Zero means not listed as present in source Table S2 and is not a new experimental demonstration of absence. Feature prevalence is the number of contexts reporting that feature divided by 15. Context-specific reporting means a feature is listed in one context. Confidence counts and retention-time distributions describe published features rather than replicate-level observations. Exact printed retention time tokens are retained alongside qualified decimal candidates for numerical display.

For two contexts, Jaccard similarity is the number of shared reported features divided by the number in their union. The service defines similarity as one if both sets are empty. The 15-context matrix is converted to distance by subtracting similarity from one. SciPy ≥ 1.11.0 (https://scipy.org/, accessed on 1 September 2026) [23] average-linkage hierarchical clustering supplies a descriptive ordering. The application also retrieves the most similar contexts for a selected profile. These calculations use binary occurrence rather than peak area. We do not recompute the published CAP analysis or perform PCA, abundance-based Bray–Curtis analysis, significance tests or multiple-testing correction on this presence layer.

### 2.6. Evidence Tracing and the Feature Context Network

Evidence Explorer retrieves a feature packet containing the exact published annotation, retention time, confidence, linked contexts, candidate interpretation, structure availability and provenance. Correction and conflict fields remain visible. The service can return a feature even when no molecular structure is assigned. This makes unknown and ambiguous records traceable without inventing chemical identities.

Feature-Context Network uses NetworkX (https://networkx.org/, accessed on 1 September 2026) [24] to create an undirected graph with typed nodes for features, contexts, source taxa, host/material labels and candidate chemical interpretations. Four allowed edge types represent context-species links, context-host links, published presence and chemical interpretation. Filters select contexts and confidence categories, and candidate-interpretation nodes can be hidden. The spring layout uses seed 42. Plotly (https://plotly.com/python/, accessed on 1 September 2026) displays node and edge types, while exported tables retain their source and evidence fields. Node size reflects documentation degree with a display cap. Connected components and edge counts describe coverage. No edge asserts attraction, repellency, natural-enemy recruitment, binding or causal ecological interaction.

### 2.7. Structural Similarity Analysis

Structural Similarity Explorer accepts a current curated candidate or bounded custom SMILES query. RDKit 2026.03.1 parsing precedes calculation. Morgan fingerprints use radius 2 and 2048 bits [25,26]. Tanimoto similarity is the number of shared set bits divided by the number in their union. Results are reported from zero to one and rounded to four decimal places by the service. Users can filter the eligible candidate set by source confidence and apply a minimum numerical similarity from zero to one, with a default of zero. A pairwise comparison uses the same fingerprints for the selected structure-enabled records. No universal high-similarity threshold is asserted. Candidate comparisons retain source Bio IDs, names and identification confidence. Structurally unresolved annotations and unknowns are excluded from structure calculations, but remain present in the feature and evidence views. Molecular similarity does not imply a similar odour, emission rate, biological response or receptor interaction.

### 2.8. References Assistant and Application Delivery

References/Data Sources identifies the primary article and distinguishes source Table S1 context metadata from source Table S2 feature evidence. It also explains the inherited confidence notation. The Research Team Assistant provides local, deterministic retrieval through guided questions and a bounded text field matched by explicit keywords or Bio identifiers. Responses use predefined templates and return an evidence layer, source, caution and supporting records. It does not use an external language-model API or generate unsupported relationships. The range of supported text questions is limited. Template content must remain synchronized with the displayed release, including the separate docking results.

The application is implemented in Python 3.10.0 (https://www.python.org/, accessed on 1 September 2026) and Streamlit 1.37.1 (https://docs.streamlit.io/, accessed on 1 September 2026) [27]. Pandas 2.3.3 (https://pandas.pydata.org/, accessed on 1 September 2026) services construct the evidence joins, Plotly 5.22.0 provides interactive charts, and NetworkX ≥ 3.2.0 provides graph layout and summaries. Docking is executed offline. The public Docking Predictions route reads finalized production, validation and provenance tables and selected structures. Its eight views cover the overview, receptor-candidate matrix, candidate explorer, receptor comparison, seed variability, poses, validation and methods. It does not execute Vina in the browser. Finalized release data, source code and query services are archived separately from the complete scientific calculation archive.

### 2.9. Aphid OBP Structures and Production Candidate Panel

A receptor-discovery audit evaluated structurally available aphid olfactory proteins and retained two experimentally determined aphid OBP3 structures for production docking: MvicOBP3 from *Megoura viciae* (PDB 4Z39; X-ray diffraction, 1.30 Å) and NribOBP3 from *Nasonovia ribisnigri* (PDB 4Z45; X-ray diffraction, 2.02 Å) [28]. These proteins are aphid OBPs but are not proteins from the 10 aphid species in the source GC-MS dataset. The historical RferOBP4 model from *Rhynchophorus ferrugineus* was retained only as historical development material and was excluded from the production results. The production ligand panel contained 20 evidence-linked candidates: all 11 authentic-standard-supported Tier A structures and nine selected Tier B structures. Tier C candidates were not included in the production screen. The panel therefore represents a deliberately bounded structure-enabled subset rather than the complete set of 81 published Bio features.

### 2.10. Receptor and Ligand Preparation

Chain A was used for both 4Z39 and 4Z45. Waters and non-protein heteroatoms were removed. Receptors were hydrogenated at an assumed pH of 7.0 with PDBFixer 1.12.0 in earlier records (https://github.com/openmm/pdbfixer) and OpenMM 8.5.1 (https://openmm.org/, accessed on 1 September 2026) [29] and converted to AutoDock-compatible PDBQT with Gasteiger charges and Meeko 0.7.1. The pH value is a standardized computational preparation condition and is not presented as a measurement of aphid sensillar-lymph pH. In 4Z45, residues 119–121 are unresolved in the crystal structure and lie outside the selected docking region. Preparation required a deterministic correction of generated terminal OXT geometry at Asp118; this correction did not alter the docking box or pocket definition.

Ligand identities and structures were finalized before docking. ETKDGv3 requested 20 conformers per ligand with seed 20,261,004. The lowest optimized-energy conformer among those successfully generated was retained. The preparation code used MMFF94s when available, with UFF as a fallback. The per-ligand preparation records report the force field actually used. Meeko 0.7.1 (https://github.com/forlilab/Meeko) then generated PDBQT files. For Bio17, Bio4 and Bio21, the source did not uniquely specify experimental stereochemistry. A coordinate realization of each constitutional candidate was used without assigning it as the experimentally identified stereoisomer.

### 2.11. Docking Site and AutoDock Vina Protocol

Neither 4Z39 nor 4Z45 contains a co-crystallized VOC. Northey et al. [28] described a surface groove as a putative ligand-binding region in these aphid OBP3 structures. Prospective docking boxes were therefore defined around documented groove residues with a 5 Å margin. For 4Z39 the box centre was (8.284, 6.721, 22.849) Å with dimensions 27.811 × 29.469 × 22.853 Å; for 4Z45 the centre was (21.128, −20.253, 1.603) Å with dimensions 22.254 × 27.693 × 26.435 Å. These boxes are literature/residue-informed computational search regions rather than experimentally validated VOC pockets.

Production docking used AutoDock Vina 1.2.7 [30,31], exhaustiveness 32, 20 requested modes, an energy range of 4 kcal/mol and one CPU thread per run. Five predefined seeds (42, 2026, 104,729, 271,828 and 314,159) were used for every receptor–ligand combination. Twenty VOC candidates × two OBPs × five seeds yielded 200 independent computational runs. Ten poses per successful run were retained, giving 2000 poses. The primary pair-level descriptor was the median rank-1 Vina docking score across the five seeds. Vina scores are reported as docking scores in kcal/mol and are not interpreted as binding free energies or Ki values.

### 2.12. Prospective Docking Validation

Before production docking, the preparation and docking workflow was evaluated by ligand redocking with a prospectively defined success threshold of symmetry-corrected heavy-atom RMSD ≤ 2.0 Å. A development system (1DQE/bombykol) and two held out systems (2WC6/bombykol and 3N7H/DEET) were evaluated with the same five-seed framework. Rank-1 recovery and best of 10 recovery were recorded separately because pose sampling and score ranking are distinct properties of a docking workflow. Control search boxes used native-ligand heavy atom bounds with a 6 Å margin on each face and a minimum dimension of 22 Å. Production boxes used the separately defined literature-informed residue bounds with a 5 Å margin. The finalized control protocol and production box definitions are supplied with the Supplementary Information.

The development control recovered a qualifying pose among the best 10 in 1/5 seeds, but never at rank 1. Held-out 2WC6 recovered a qualifying pose among the best 10 in 5/5 seeds and at rank 1 in 1/5. Held-out 3N7H recovered the crystal pose at rank 1 in 5/5 seeds. These controls support exploratory pose sampling. They also show that score ranking is system-dependent. They do not validate predicted affinity or the binding region of either production aphid OBP.

### 2.13. Docking Reproducibility and Statistical Summaries

All 40 production pairs were summarized across five seeds. We retained the sample standard deviation, score range and pose/contact consistency. The post hoc descriptive stability rule required all five seeds, score SD ≤ 0.20 kcal/mol, mean pairwise residue-contact Jaccard similarity ≥0.60, and a largest ligand–centroid cluster containing at least three seeds at a 3 Å threshold. This rule classified 37 pairs as stable and three as variable. The thresholds are computational diagnostics, not biological validation criteria. Pearson correlation compared the two 20-ligand median-score profiles. Spearman correlation with heavy-atom count screened for possible molecular-size scoring bias.

Representative complexes were selected for visualization from stable pairs. For each OBP, the selection considered the most negative Tier A median, the largest receptor contrast in that OBP’s direction, and a chemically distinct candidate. The diversity criterion used Morgan fingerprints with radius 2 and 2048 bits. For each selected pair, the displayed pose was the rank-1 pose with a score closest to its five-seed median. Ties retained the first matching row in the finalized run table. The complete selection record is included in the Supplementary Data. These poses illustrate predictions and are not independently validated binding modes.

### 2.14. Platform Consistency and Release Verification

We compared the archived route definitions and active query services with the supplied interface captures and audit records. Identifier checks assessed uniqueness, consistency of feature–chemical mappings, and whether all feature and biological-context references corresponded to valid records. We independently reconstructed the binary presence matrix directly from the finalized observation table and compared the resulting summary statistics with those generated by the platform. Full-context network tables were regenerated from the archived services. Count reconciliation and source-confidence preservation test the data-integration hypothesis. These checks assess data consistency and implemented functionality. They do not replace a usability study, live-deployment verification or new experimental validation. Supplied structure-validation and docking audits are distinguished from calculations independently repeated for the platform consistency checks.

## 3. Results

### 3.1. Reconstructed Published Evidence and Structure-Enabled Chemical Layer

The evidence layer contains all 81 published Bio features across 15 contexts. It retains 11 authentic-standard-supported annotations, 32 higher-confidence tentative library identifications, 27 lower-confidence tentative library identifications and 11 unknown features. Seventy features have named candidate interpretations. Fifty-five have curated molecular representations, while 15 named interpretations remain structurally unresolved. The unknown records have no inferred chemical structures. The independent matrix reconstruction matched the statistics service exactly. Feature and context identifiers were unique. The feature–chemical mapping was unique by feature, and no dangling feature or context links were found in the checked presence records.

Supplementary Table S1 preserves the published feature annotations. Table S2 lists the 20 production ligands with identifiers and source confidence. Tables S3–S9 document preparation, search boxes, redocking controls, pair summaries, stability, corrected contacts and stereochemistry. Table S10 records the published sample contexts. The complete 55-structure table is supplied as Supplementary Table S11 and in the machine-readable data package.

### 3.2. Dashboard and Published Feature Catalogue

**Figure 2:**
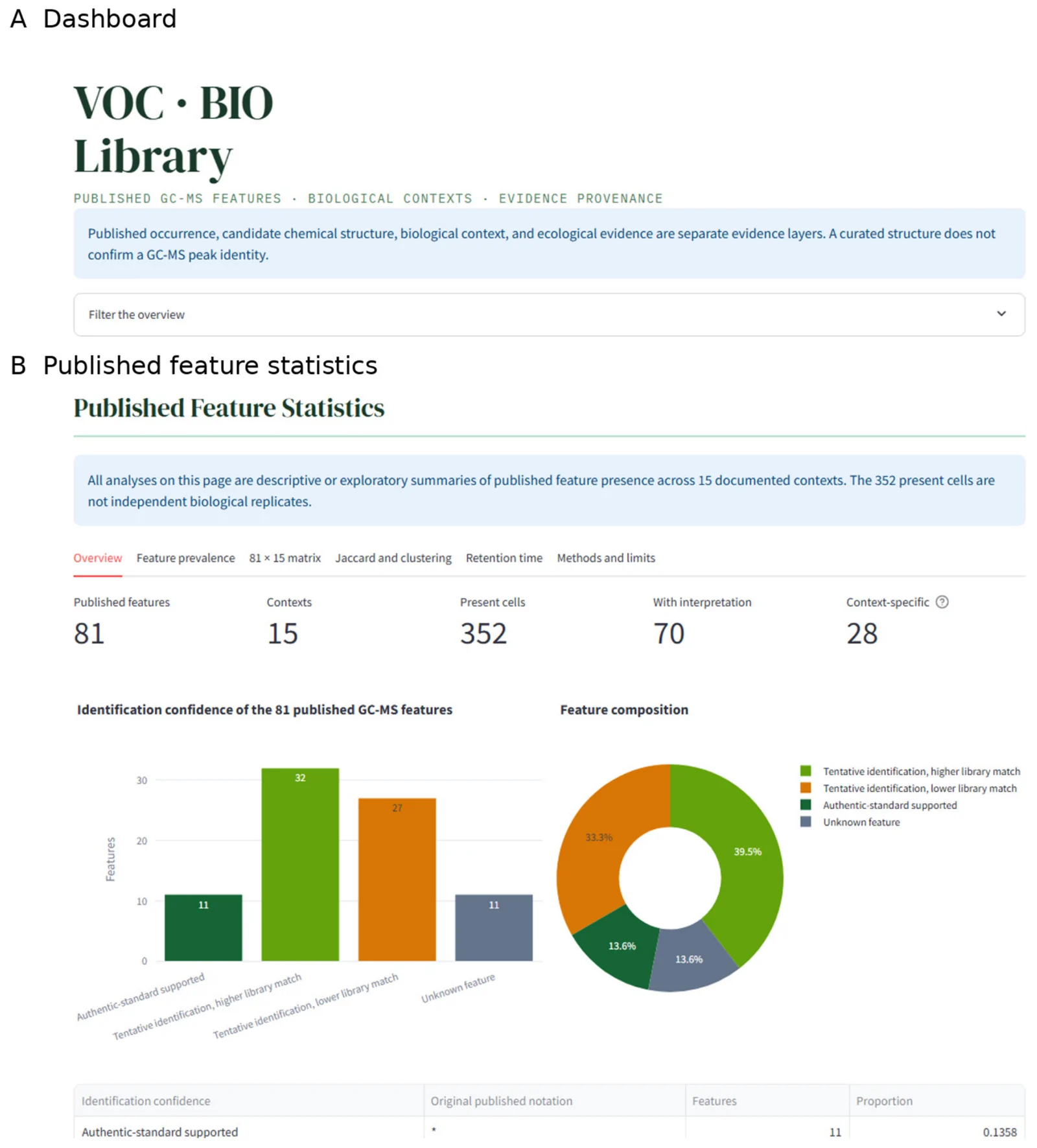
Representative VOC·BIO Library Version 1.0 interface. (**A**) The dashboard presents the evidence-layer design and available research routes. (**B**) Published Feature Statistics summarizes the 81 GC-MS features across 15 documented contexts and preserves the published identification-confidence categories. The interface separates published occurrence, candidate chemical interpretation and curated molecular structure.

**Figure 3:**
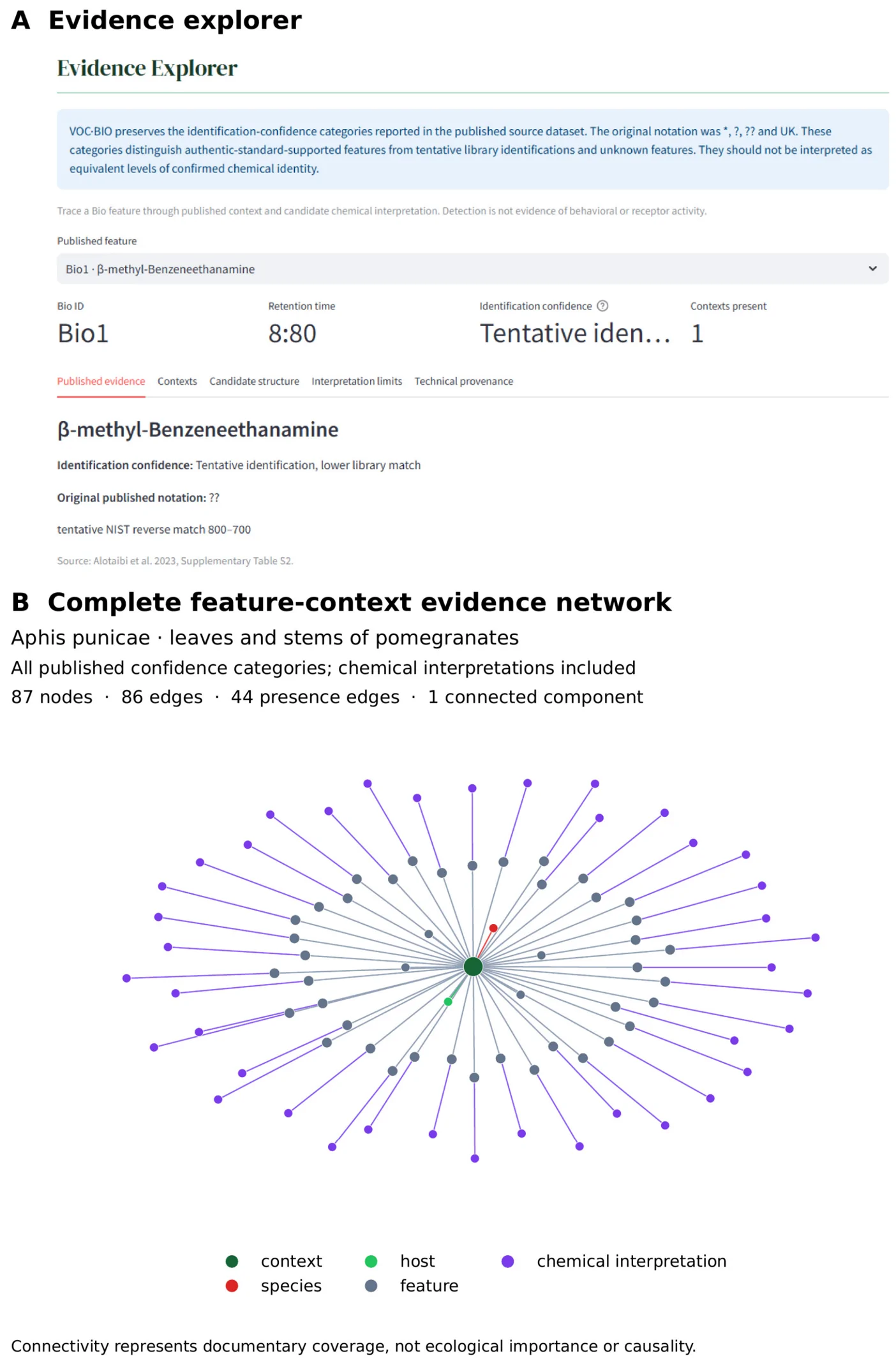
Selected interface regions from the evidence explorer (**A**) and feature-context network (**B**). Additional analytical modules are shown in Supplementary Figures S2 and S3. Screenshots were cropped for readability; original screenshot colours are retained. The ellipsis in panel (**A**) abbreviates the lower-match tentative-identification label; the full category is defined in Section 2.1. Panel (**B**) shows a cropped portion of the selected-context graph, not the complete network. Dark green nodes denote contexts, red nodes taxa, bright green nodes host/material records, grey nodes published features and purple nodes chemical interpretations. Grey edges encode published presence, purple edges chemical interpretations, red edges context–taxon links and green edges context–host links.

All 11 main routes are defined in the archived application. Their inputs and computational roles are summarized in Table 1. The unfiltered Dashboard reports 81 features, 15 contexts, 352 presence records, 70 interpretations, 55 candidate structures and 11 unknown features. Published GC-MS Features supplies a feature-level entry point and keeps unknowns and unresolved annotations visible. Filtering by biological context returns the linked unique features without multiplying the feature count by the number of occurrence rows. Figure 2 and Figure 3 show representative interface views. Supplementary Figures S1–S3 cover the remaining routes.

### 3.3. Biological and Sample Context Exploration

The context catalogue exposes 15 published sample/host-location records representing 10 source-publication taxon labels. It retains accession, host/material, date, locality and environmental metadata. Context profiles contain between 8 and 44 reported features. The pomegranate context has 44 reported features, while the radish context has eight. These totals describe documentary reporting across the published presence table. They do not show that one taxon produces more volatile compounds than another. The foxtail, feed and Alhada conflicts remain visible and limit comparisons that depend on those labels.

### 3.4. Candidate Structures and Chemical Properties

Chemical Structure Explorer makes 55 candidate structures available across all three named source-confidence groups. The layer includes all 11 authentic-standard-supported annotations, 30 higher-confidence tentative annotations and 14 lower-confidence tentative annotations. Each eligible candidate has a linked structure representation and identifier record. The supplied structure audit reports RDKit parsing and identifier checks. Those checks establish an internally usable molecular representation, not independent experimental confirmation of the peak. Descriptor and depiction views allow researchers to examine physicochemical differences before selecting structures for comparison or docking. The 26 records without a resolved structure remain accessible through the feature catalogue and Evidence Explorer.

### 3.5. Descriptive Feature Statistics and Context Comparisons

**Figure 4:**
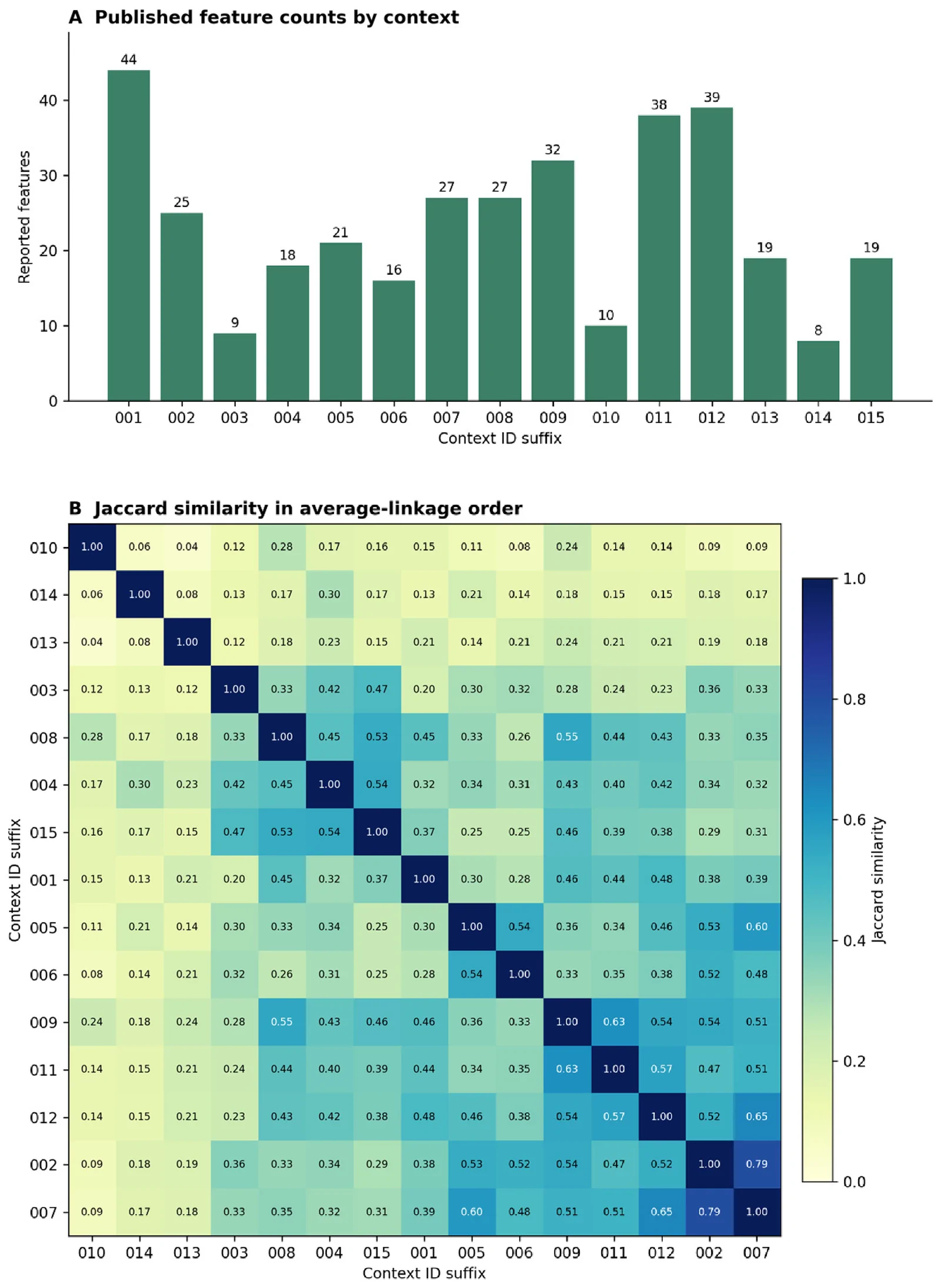
Descriptive summaries of published feature-context presence. (**A**) Number of reported features in each of the 15 contexts. (**B**) Jaccard similarity between their binary presence profiles, shown in average-linkage order. The matrix contains 81 features and 352 reported presence cells. Zero means not listed as present. These summaries do not use peak areas or independent biological replicates.

The independently reconstructed 81 × 15 matrix contains 352 reported presence cells. Twenty-eight features occur in only one documented context. Bio30, annotated as 4,5-dimethyl-nonane, is reported in 14 contexts, the highest prevalence in this layer. Bio14, Bio50, Bio56 and Bio68 are each reported in 13 contexts. Their shared reporting does not establish common emission origin or equivalent identification confidence. Figure 4 summarizes context counts and pairwise similarity. The numerical results and feature-prevalence table accompany the supplement.

Off-diagonal context Jaccard values range from 0.0357 to 0.7931. The highest value is between CTX-V1-002 and CTX-V1-007, with 23 shared features in a union of 29. The lowest is between CTX-V1-010 and CTX-V1-013, with one shared feature in a union of 28. Average-linkage clustering orders these documentary profiles for inspection. The ordering is exploratory and is not a validated species classifier. No significance or biological effect size is assigned to these similarities.

### 3.6. Evidence Explorer and Feature Context Network

Evidence Explorer links every Bio record to its source annotation and contexts and adds a candidate interpretation where available. The full-context network, with chemical interpretations enabled, contains 190 nodes: 81 features, 70 interpretations, 15 contexts, 10 taxon labels and 14 host/material labels. It contains 452 edges: 352 published presence links, 70 interpretation links and 15 links each for context-species and context-host metadata. The 14 host/material nodes follow exact labels and are not 14 independently curated botanical species. These complete network counts differ from a filtered interface view. They measure documented coverage rather than ecological importance. The feature-to-candidate links let users move between biological reporting and molecular representations without losing the source confidence. Unknown features remain graph nodes through their presence links. The network has no behavioural-effect or natural-enemy edge type. It therefore provides an evidence map rather than a demonstrated ecological interaction model.

### 3.7. Structural Similarity Exploration

The similarity service compares the 55 structure-enabled candidate records or a confidence-filtered subset. It accepts a library candidate or a valid bounded SMILES input. Ranked output retains the Bio identifier, candidate name, numerical Tanimoto coefficient and source confidence. The same fingerprint settings define the pairwise comparison matrix. This provides a reproducible way to inspect structural neighbourhoods and choose chemically different candidates. The present study does not benchmark similarity against odour, binding or behavioural measurements. A similar molecular fingerprint therefore remains a structural result.

### 3.8. References and Research Team Assistant

The References/Data Sources view identifies the source publication and the distinct roles of its two Supplementary Tables. It also preserves the original confidence definitions. The Research Team Assistant returns supported feature, confidence, structure, mass and provenance answers with an evidence label and caution. Its text field routes a limited set of keywords and Bio identifiers to local templates. It is not an unrestricted scientific question-answering system. Inspection identified a release-synchronization limitation in the docking template, which retains a statement from before the finalized production analysis. Docking values should be read from the dedicated Docking Predictions route.

### 3.9. Docking Validation

**Figure 5:**
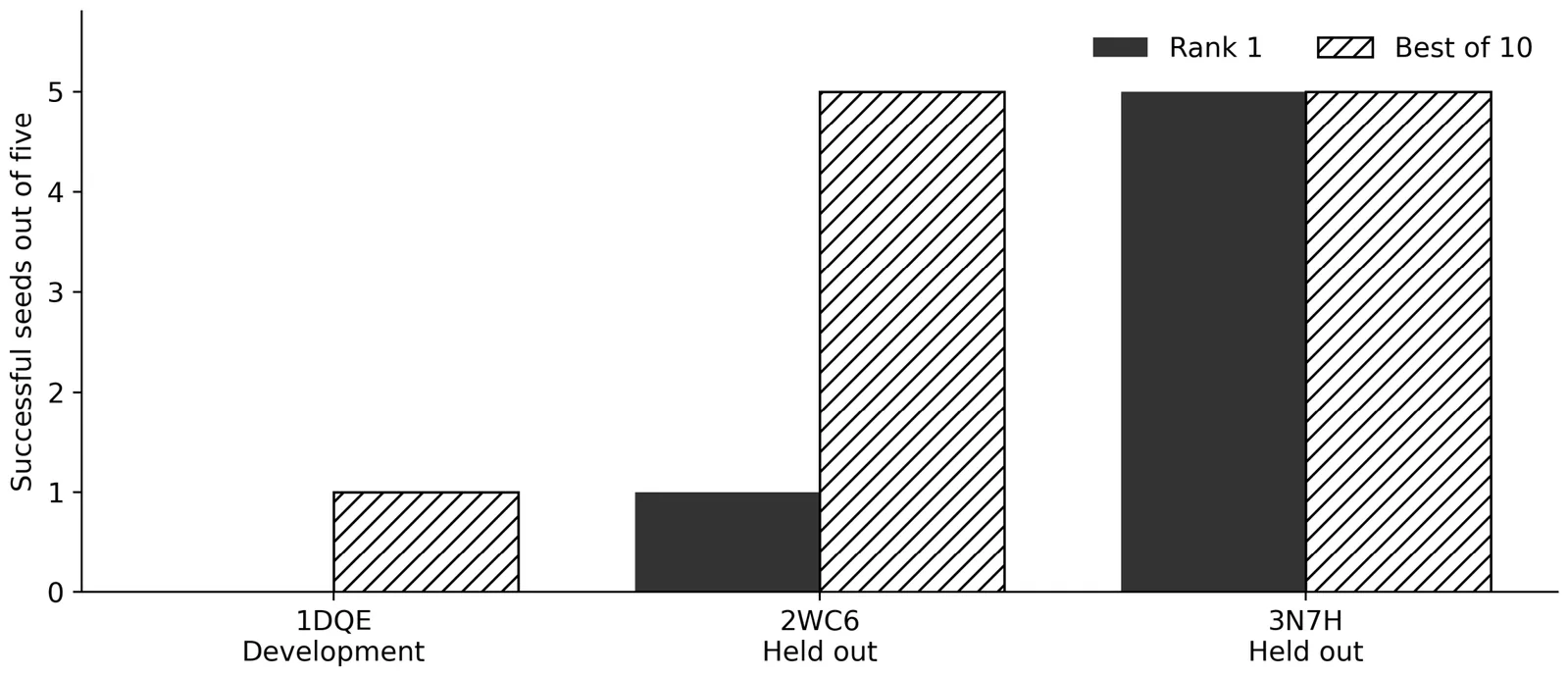
Prospective docking validation. Success indicates a symmetry-corrected heavy-atom RMSD ≤ 2.0 Å relative to the crystallographic ligand pose. The controls support exploratory pose sampling but show receptor-dependent ranking performance.

The redocking controls showed that pose sampling and Vina ranking were not equally reliable across systems. The development 1DQE control gave 0/5 rank-1 successes and 1/5 best-of-10 successes (minimum observed RMSD 1.794 Å). Held-out 2WC6 gave 1/5 rank-1 and 5/5 best-of-10 successes (median best-of-10 RMSD 1.344 Å), whereas held-out 3N7H gave 5/5 rank-1 and 5/5 best-of-10 successes (median rank-1 RMSD 0.645 Å). These results qualified the workflow for exploratory production docking while requiring multiple poses, repeated seeds and cautious interpretation of score ranking. Figure 5 summarizes these control results.

### 3.10. Multi OBP Production Docking

**Figure 6:**
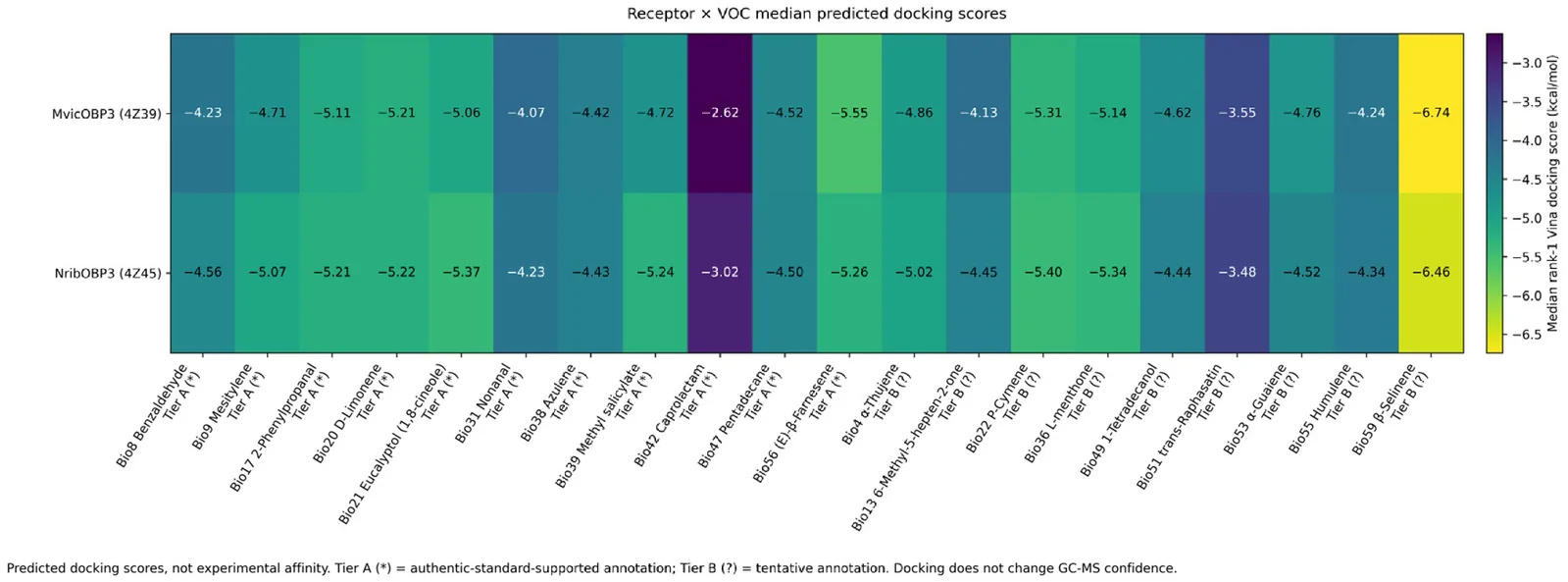
Median rank-1 AutoDock Vina docking scores across five seeds for the 20 VOC candidates and two aphid OBPs. Values are computational docking scores (kcal/mol), not measured affinities.

All 200 predefined production runs completed successfully and yielded 2000 retained poses. For MvicOBP3, the median of the 20 pair medians was −4.712 kcal/mol, with pair medians ranging from −6.739 to −2.624 kcal/mol. For NribOBP3, the corresponding median was −4.7895 kcal/mol, with a range from −6.461 to −3.016 kcal/mol (Figure 6).

Bio59, annotated as beta-selinene in the source evidence, had the most negative pair median for both OBPs (−6.739 kcal/mol for MvicOBP3 and −6.461 kcal/mol for NribOBP3). Bio42, caprolactam, had the least negative pair median for both (−2.624 and −3.016 kcal/mol, respectively). These extrema describe the computational score distribution within the screened panel and are not evidence that beta-selinene is the strongest biological binder or that caprolactam is the weakest biological ligand.

**Figure 7:**
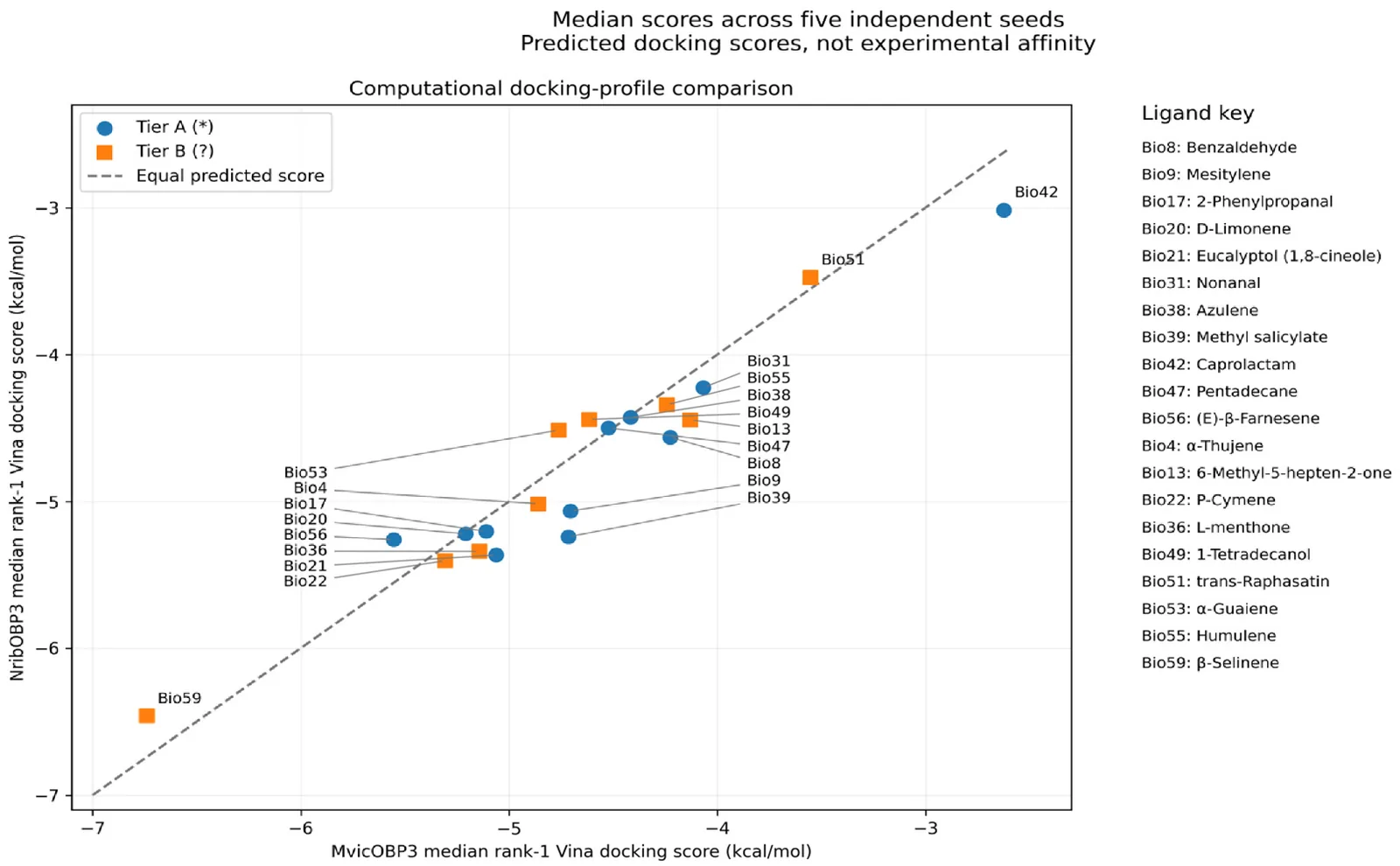
Comparison of median docking-score profiles for MvicOBP3 and NribOBP3 across the 20-candidate VOC panel. Each point represents the median rank-1 AutoDock Vina score across five independent seeds. Blue circles denote Tier A candidates, whose source GC-MS annotations were supported by authentic standards (*). Orange squares denote Tier B candidates, whose source annotations were tentative library identifications (?, reported NIST reverse match 990–850). These symbols describe published identification confidence, not statistical significance or docking validation. Pearson *r* = 0.9600 (*n* = 20). The dashed line indicates equal predicted scores. The correlation describes similarity of computational score profiles and does not imply a shared biological response.

The two 20-ligand median-score profiles were strongly correlated (Pearson *r* = 0.9600, *n* = 20; Figure 7). This describes similar Vina score patterns under the chosen protocol. Bio39, methyl salicylate, had the largest descriptive receptor contrast. Its NribOBP3 median was 0.525 kcal/mol more negative than its MvicOBP3 median. This difference does not establish experimental receptor preference.

### 3.11. Seed Variability and Interaction Checks

Thirty-seven of the 40 receptor–ligand pairs met the uniform post hoc descriptive stability rule. Three pairs were flagged as variable: MvicOBP3-Bio42, NribOBP3-Bio49 and NribOBP3-Bio56. The MvicOBP3-Bio42 pair showed pose/contact inconsistency despite a narrow score spread, whereas NribOBP3-Bio49 and NribOBP3-Bio56 showed larger across-seed score variation. These flags identify computational sensitivity and are not measures of biological instability. Interaction-level audit corrected two heavy-atom distances in the representative NribOBP3-Bio39 pose to 2.905 Å between ARG4 N and ligand O and 3.591 Å between TYR30 CB and ligand O. Meeko ring-closure dummy atoms were excluded from corrected Bio38 geometry and pose schematics without changing docking scores or stability classification.

## 4. Discussion

VOC·BIO Library brings the published feature record, biological context and complementary computations into one interface. The 11 features have distinct roles: the Dashboard establishes scope; the feature catalogue provides access; context views explain where records were reported; chemistry and similarity operate on candidate structures; statistics summarize occurrence; evidence and network views expose traceable links; references identify source roles; the assistant provides guided retrieval; and docking adds a bounded molecular prediction layer. None replaces the others. This organization allows a researcher to follow a Bio feature across the platform while seeing which evidence supports each representation.

The count and join checks support the proposed data-integration hypothesis within this finalized dataset. All 81 features remain represented, including the 11 unknowns and 15 named interpretations without resolved structures. The binary matrix independently reconstructed from the source CSV matched the platform-generated summary statistics, and all checked feature and context references corresponded to valid records. These findings demonstrate consistency of the current evidence representation. They do not establish universal software reliability or validate the underlying chemical annotations. The outdated assistant docking template shows why cross-view content must also be checked whenever a release changes.

The descriptive statistics and context network extend the value of the platform beyond individual molecule inspection. Twenty-eight features are listed in one context, while several are reported broadly. Such patterns can guide which records deserve closer examination. They cannot by themselves identify a diagnostic volatile or explain differences among taxa. Source contexts combine taxon, host material, locality and collection setting. The available published table does not isolate their effects. A feature can also be host-associated or have uncertain origin. Confidence-aware views help prevent widely reported tentative annotations from being mistaken for broadly confirmed compounds.

Chemical descriptors and structural similarity provide a different form of exploration. Connecting molecular properties to biological records remains useful, but a structure is only a representation of a named interpretation until peak identity is experimentally supported. The 55 curated structures permit consistent calculations and comparison. They do not increase the 11-feature authentic-standard-supported subset. The fingerprint settings and user selected threshold make structural searches explicit. Their usefulness for predicting odour or insect response remains to be tested. Exact mass, LogP and TPSA also do not provide a validated substitute for measured vapour pressure or Henry’s law constants. The descriptor radar is therefore presented as a display of molecular properties.

The network and deterministic assistant serve evidence navigation. A documented presence edge means that a feature was listed in a source context. It does not mean that the feature attracts or repels that insect. Similarly, an assistant response is a template-based retrieval result with a defined evidence scope. It is not independent scientific reasoning or a language-model-generated interpretation. This local design supports reproducibility and avoids an external model dependency for answers. PubChem depictions still require external access, while local structure calculations require RDKit. A versioned update should synchronize the assistant docking wording with the finalized production route before a live release is represented as fully consistent.

VOC·BIO Library complements resources such as PheroBase, mVOC, GNPS and MACE [17,18,19,20]. Their semiochemical, microbial-volatile, mass-spectrometry and spectral-reference functions have different scopes. VOC·BIO has a narrow regional source dataset and does not claim comparable coverage. Its role is to connect a defined aphid evidence set to context summaries, structure-enabled analysis and inspectable provenance. The platform also keeps source evidence, candidate interpretation and locally generated predictions apart. This distinction is central when integrating literature records with computations.

The docking study demonstrates one additional analytical use of the structure layer. Twenty candidates were evaluated against two experimentally determined aphid OBP3 structures. MvicOBP3 and NribOBP3 are not membrane odorant receptors, and neither belongs to the 10 source GC-MS taxa [28]. The held-out controls show that successful pose sampling does not guarantee reliable rank-1 scoring. Repeated seeds and pose/contact diagnostics therefore accompany the reported medians. The strong correlation between the two score profiles is a computational pattern under this protocol. The moderate association of MvicOBP3 score with ligand heavy-atom count (Spearman rho = −0.491, *n* = 20) suggests a possible size contribution. Neither the correlation nor the most negative score establishes biological ligand preference.

The study has several limits. It begins with one regionally restricted experimental publication. The surviving workbooks do not fully reconstruct the published 63-observation × 81-feature quantitative analysis. Peak-level spectra, all standard comparisons and blank/contamination records are not linked to every feature in the reconstructed archive. Published confidence is therefore retained rather than revalidated. The exact emission origin of each feature is also unresolved. The foxtail/feed locality conflicts and Alhada taxonomic conflict remain documentary limitations. Their presence should qualify any context-dependent interpretation.

Docking covers only 20 candidates and two aphid OBPs. The production structures lack co-crystallized VOCs, so their search regions are literature-informed surface grooves rather than experimentally confirmed VOC pockets. Five seeds are computational repeats, not biological replicates. Three candidate structures retain source stereochemistry uncertainty. Complete preparation records, raw poses, logs and validation execution assets are supplied in the accompanying frozen docking reproducibility archive, separately from the software release identified by the Zenodo DOI. Supplied interface captures and local checks do not independently establish the state of the live application.

Future development should strengthen the evidence represented by all modules. Priorities include additional curated datasets, peak-to-spectrum and standard links, resolved sample metadata, functional assays and broader olfactory-protein coverage. Measured volatility, binding, electrophysiological and behavioural observations could then support appropriate tests of computational predictions. A usability study could evaluate navigation and interpretation across the 11 features. Predictive modelling should follow sufficient experimentally validated records and independent evaluation. Stable identifiers, preserved source versions and clearly separated evidence layers provide a basis for these additions without silently changing the finalized results.

## 5. Conclusions

VOC·BIO Library Version 1.0 integrates 81 published aphid GC-MS features, 15 biological contexts and 352 reported presence records through 11 research features. The platform connects the source feature catalogue to sample metadata, 55 candidate structures, molecular descriptors, structural similarity, descriptive statistics, evidence tracing, networks, references and guided retrieval. Count reconciliation and identifier checks support the consistency of these linked views. A separately benchmarked exploratory docking study adds predictions for 20 candidates and two aphid OBPs. The library retains the original identification-confidence hierarchy and unresolved evidence. Its computational outputs support further investigation but do not establish compound origin, affinity, behaviour or pest-control efficacy. Expansion should prioritize stronger source provenance, functional validation and synchronized versioned releases across the full platform.

## Figures and Tables

**Table 1:** The 11 VOC·BIO Library features and their implemented computational roles. Counts refer to the unfiltered finalized evidence layer.

Feature	Principal Input	Implemented Operation
Dashboard	Linked evidence tables	Counts, confidence composition and reported prevalence
Published GC-MS Features	81 source features	Search, context/confidence filters and CSV export
Chemical Structure Explorer	55 candidate structures	Identifiers, depictions and 10 molecular descriptors
Biological & Sample Contexts	15 source contexts	Metadata filtering and species-host summaries
Published Feature Statistics	352 presence assertions	81 × 15 matrix, Jaccard similarity and clustering
Evidence Explorer	Feature-context-chemical joins	Source annotation, confidence and provenance packet
Docking Predictions	Finalized offline analysis	40 pairs, five seeds, scores, poses and validation
Feature-Context Network	Typed evidence links	Graph construction, degree and component summaries
Structural Similarity Explorer	Structure-enabled candidates	Morgan/Tanimoto searches and pairwise comparison
References/Data Sources	Primary article and supplement	Source-table roles and confidence definitions
Research Team Assistant	Supported record queries	Deterministic templates with sources and cautions

## Data Availability

The primary experimental source is Alotaibi et al. [13] and its Supplementary Information. VOC·BIO Library v1.0.0 is archived at https://doi.org/10.5281/zenodo.23170512. The source repository is https://github.com/Taif-ChemEcoLab/Taif-aphid-voc-library (accessed on 1 September 2026). Original software code is released under the MIT License. DATA_LICENSE.md governs source-derived and third-party scientific material separately. The software archive includes the read-only application, linked V1 CSV evidence tables, query services, finalized docking summaries and selected display structures. The active V1 evidence layer is CSV-based; historical SQLite and parser outputs are not the scientific source used for the reported summaries. The Supplementary Data Package provides complete feature, context, chemical and analysis tables. An accompanying execution-level reproducibility archive (VOCBIO_V1_Docking_Reproducibility_FINAL.zip) is supplied within VOCBIO_Archive_Reconciled_Complete_Package.Zip as Supplementary Material. It contains receptor and ligand preparation assets, 200 production docking configurations, 200 raw AutoDock Vina outputs, 200 run logs and 2000 retained pose files. It also includes execution assets for the three control systems, finalized result tables, correction records, provenance manifests and file-level checksums. This archive complements the versioned VOC·BIO Library v1.0.0 software release at Zenodo (DOI: 10.5281/zenodo.23170512). Additional unpublished GC-MS records remain subject to applicable source and institutional data-sharing terms. Future changes should use explicit release versions with documented source and curation changes.

## Supplementary Materials

The following supporting information can be downloaded at: https://www.mdpi.com/article/10.3390/insects17101062/s1. Supplementary Methods S1–S6 describe the evidence layers, preparation and docking, controls and variability, historical reconstruction, platform computations and consistency checks. Table S1. Published 81-feature evidence layer. Table S2. Frozen production ligand panel. Table S3. Receptor provenance and preparation. Table S4. Production search boxes. Table S5. Independent redocking control summary. Table S6. Complete production pair summaries. Table S7. Post hoc stability diagnostics. Table S8. Corrected representative contacts. Table S9. Source stereochemistry caveats. Table S10. Published sample-context metadata. Table S11. Complete frozen structure-enabled candidate list. Table S12. Computational roles of all 11 platform features. Figure S1. Core application views. Published features, contexts and provenance remain separate from candidate structures. Figure S2. Evidence, cheminformatics and network views. These descriptive displays do not confirm chemical identity or ecological causality. Figure S3. Reference, guided-query and docking-provenance views from the supplied Version 1.0 interface captures. The current docking route is separate from the assistant template limitation described in Supplementary Methods S5. Figure S4. Sample SD of rank-1 scores across five seeds. Values are computational variability, not biological variance. Figure S5. Selected five-seed score distributions. Seeds are computational repeats. Figure S6. Representative MvicOBP3 poses selected by the documented rule. Dummy ring-closure atoms are excluded. Predicted geometry does not establish a binding mode. Figure S7. Representative NribOBP3 poses. The corrected heavy-atom contact annotations are in Table S8 and the full CSV. The supplementary data package contains the complete feature, context, structure and analysis tables. VOCBIO_V1_Docking_Reproducibility_FINAL.zip contains the frozen execution-level docking assets and checksums. The reference was used in supplementary document is [13].

## Abbreviations

VOC, volatile organic compound; GC-MS, gas chromatography-mass spectrometry; OBP, odorant-binding protein; OR, odorant receptor; CAP, canonical analysis of principal coordinates; SMILES, simplified molecular-input line-entry system; RMSD, root-mean-square deviation; PDB, Protein Data Bank.
