# Supplementary material for: VOC·BIO Library: An Integrated Platform for Published Aphid GC-MS Evidence, Biological Contexts, Cheminformatics and Exploratory OBP Docking
Source: Insects. 2026 Oct 15;17(10):1062. doi: 10.3390/insects17101062 (PMC13705314; doi:10.3390/insects17101062)
Supplement: Supplementary file 1 [file insects-17-01062-s001.zip › VOCBIO_Archive_Reconciled_Complete_Package.zip!/VOCBIO_Balanced_Figure_Exports.zip!/VOCBIO_Balanced_Figure_Exports/Figure4_context_evidence.pdf]

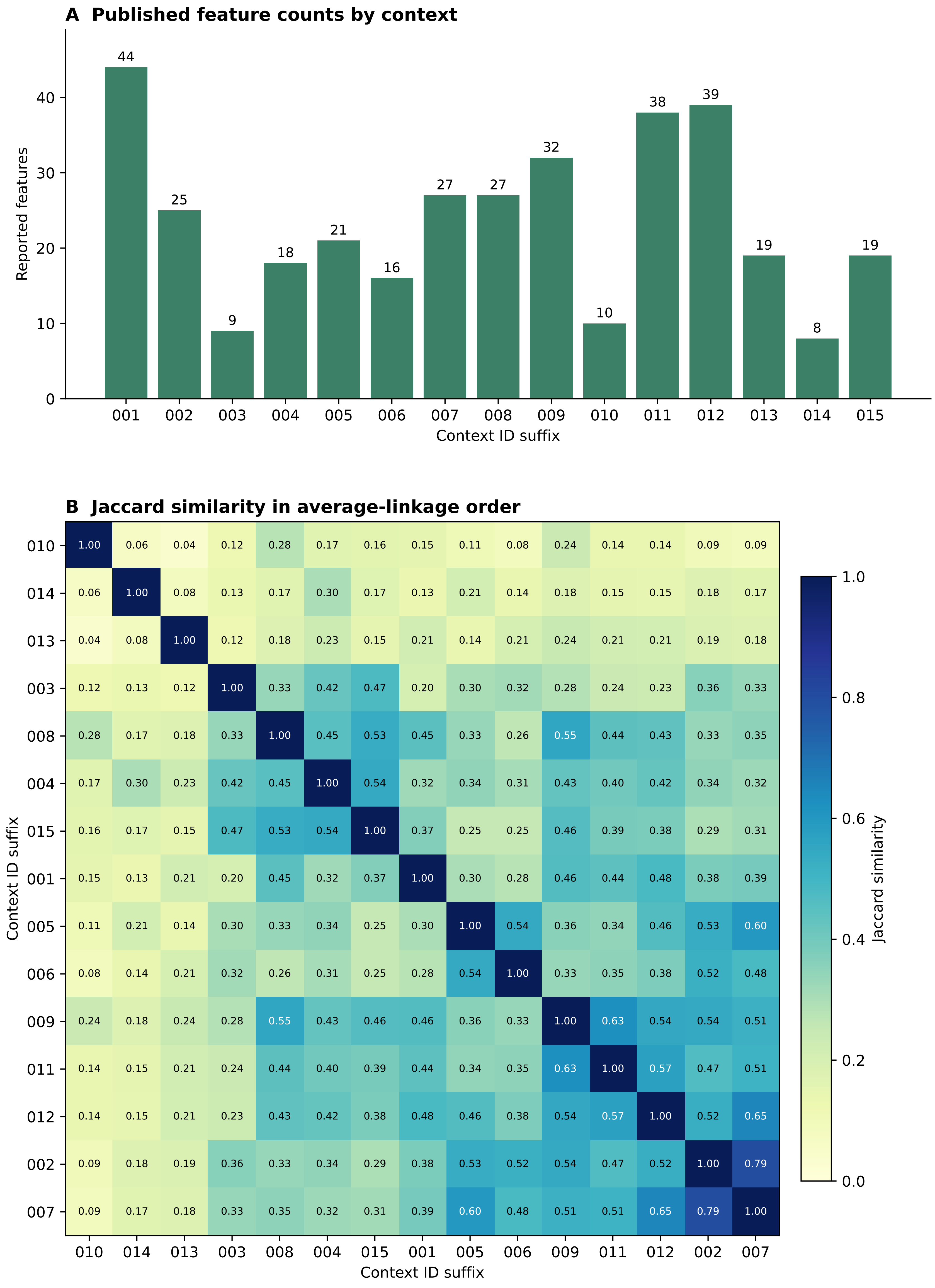
A Published feature counts by context
Reported features
Context ID suffix
44
25
9
18
21
16
27
27
32
10
38
39
19
8
19
001
002
003
004
005
006
007
008
009
010
011
012
013
014
015
B Jaccard similarity in average-linkage order
Context ID suffix
Jaccard similarity
010
014
013
003
008
004
015
001
005
006
009
011
012
002
007
