# Supplementary material for: VOC·BIO Library: An Integrated Platform for Published Aphid GC-MS Evidence, Biological Contexts, Cheminformatics and Exploratory OBP Docking
Source: Insects. 2026 Oct 15;17(10):1062. doi: 10.3390/insects17101062 (PMC13705314; doi:10.3390/insects17101062)
Supplement: Supplementary file 1 [file insects-17-01062-s001.zip › VOCBIO_Archive_Reconciled_Complete_Package.zip!/VOCBIO_Balanced_Figure_Exports.zip!/VOCBIO_Balanced_Figure_Exports/Figure1.pdf]

VOC·BIO Library links 11 research features

Linked source and candidate evidence

81 published features • 15 contexts • 352 presence records

70 named interpretations • 55 candidate structures

Evidence and context

Dashboard
Scope, confidence and coverage

Published GC-MS Features
Search, filters and annotations

Biological & Sample Contexts
Metadata and context profiles

Published Feature Statistics
Presence, Jaccard and clustering

Evidence Explorer
Feature provenance and conflicts

Feature-Context Network
Typed documentation links

Chemistry and research tools

Chemical Structure Explorer
Candidate depictions and descriptors

Structural Similarity Explorer
Morgan fingerprints and Tanimoto

Docking Predictions
Frozen scores, poses and controls

References / Data Sources
Sources and confidence definitions

Research Team Assistant
Templates with supporting records

Docking: 20 candidates × 2 aphid OBPs

200 runs and 2000 retained poses

Shared interpretation boundary

Source confidence is retained. Presence is documentary.
Candidate structures do not confirm peak identities. Computations do not establish affinity, behaviour or ecological function.
