# Supplementary material for: VOC·BIO Library: An Integrated Platform for Published Aphid GC-MS Evidence, Biological Contexts, Cheminformatics and Exploratory OBP Docking
Source: Insects. 2026 Oct 15;17(10):1062. doi: 10.3390/insects17101062 (PMC13705314; doi:10.3390/insects17101062)
Supplement: Supplementary file 1 [file insects-17-01062-s001.zip › VOCBIO_Archive_Reconciled_Complete_Package.zip!/VOCBIO_Supplement_Archive_Reconciled.pdf]

Supplementary Information

VOC·BIO Library published evidence context analyses cheminformatics and exploratory docking

This supplement accompanies manuscript insects-4583511 and describes the evidence and computations used across all 11 VOC·BIO Library features. It separates published GC-MS records, candidate structures, documentary context analyses and exploratory docking. Tables S1-S11 retain the existing feature, chemistry and docking evidence. Table S12 documents the complete platform feature-method crosswalk. Complete fields and the newly regenerated presence, prevalence, similarity and network tables are supplied in VOCBIO_Archive_Reconciled_Complete_Package.Zip, within VOCBIO_Balanced_Supplementary_Data.zip. Presence records are not calibrated concentrations or independent biological replicates.

Supplementary Methods S1 Evidence and chemical interpretation

The evidence layer reproduces Table S2 of Alotaibi et al. (2023; doi:10.3390/insects14070589). The 81 features comprise 11 features supported by authentic standards, 32 higher-confidence tentative library identifications, 27 lower-confidence tentative library identifications and 11 unknown features. The original notation is preserved in the tables for traceability. An asterisk (*) denotes authentic-standard comparison. One question mark (?) denotes a tentative NIST reverse match of 990-850. Two question marks (??) denote a tentative reverse match of 800-700. UK denotes an unknown feature with a reverse match below 700. These are the source confidence categories. They were not independently reassigned. A resolved candidate structure does not upgrade source identification confidence. Published retention-time punctuation is preserved. Decimal interpretations used in comparisons remain explicitly qualified.

Table S1 of this supplement is distinct from the source publication's Table S1. Our Table S1 lists the 81 chemical features. Our Table S10 lists the published sample metadata. The accompanying published_feature_context_presence.csv retains all 15 context-occurrence columns. Peak areas, absolute concentrations and emission origins are unavailable at this publication-feature level. They are not reconstructed by inference. Printed occurrence totals that conflict with source presence cells remain documentary discrepancies.

Supplementary Methods S2 Preparation and descriptive analysis

Production used chain A of 4Z39 and 4Z45. Receptor preparation removed waters and non-protein heteroatoms. Hydrogenation assumed pH 7.0. PDBFixer/OpenMM and Meeko generated typed PDBQT files with Gasteiger charges. Unresolved 4Z45 residues Lys119-Thr121 were not modeled. Only a generated terminal OXT coordinate at Asp118 was repositioned after a valence-sanitization failure. The correction left experimental atoms and pocket definitions unchanged. This is a technical preparation correction, not a measured protonation state.

Ligand preparation requested 20 ETKDGv3 conformers with seed 20,261,004. The lowest optimized-energy conformer among those generated was retained. MMFF94s was used when available, with a UFF fallback in the code. The supplied preparation audit records the force field and conformer count for each ligand. The source stereochemistry caveats for Bio17, Bio4 and Bio21 remain unchanged.

Vina 1.2.7 used exhaustiveness 32, 20 requested modes, an energy range of 4 kcal/mol and one CPU thread. Five seeds were used: 42, 2026, 104,729, 271,828 and 314,159. Ten poses per run were retained. Forty pairs therefore produced 200 seed-level runs and 2000 retained poses. The pair descriptor is the median rank-1 Vina score, not affinity.

The post hoc stability screen required five completed seeds, sample score SD ≤0.20 kcal/mol, mean pairwise residue-contact Jaccard similarity ≥0.60, and a largest centroid cluster containing at least three seeds at 3 Å.

The rule is descriptive. It classified 37 pairs as stable and three as variable. Dummy ring-closure atoms were excluded from the corrected ligand geometry.

For representative complexes, stable pairs were selected using Tier A median score, descriptive receptor contrast and chemical diversity. Morgan fingerprints used radius 2 and 2048 bits for the diversity step. The representative pose was the rank-1 pose closest to the pair's five-seed median. A tied value retained the first matching row in the frozen run table. Contact categories are geometric screens. Polar proximity does not prove a hydrogen bond, and aromatic proximity does not prove a π interaction without angular validation.

Supplementary Methods S3 Frozen protocol and box definitions

The control protocol phase5d2_protocol_v1.0 was finalized at 2026-10-03T23:22:09.082232+00:00. Its recorded SHA-256 is 0a80027f93fd7450d942ab98ce9fba1a70c743996d13e18887fd45725fd9fc19. This is the canonical JSON hash before the protocol_sha256 field is added, not the byte hash of the final JSON file. The canonical hash was reproduced during this document audit.

Development compared native-ligand box margins of 6 Å and 4 Å. The 6 Å variant was retained because it recovered one qualifying pose among the top ten in one of five seeds; neither variant passed rank-1 recovery. The protocol was frozen before held-out evaluation. Control boxes used native-ligand heavy-atom bounds plus 6 Å on each face, with a minimum dimension of 22 Å. Production boxes used heavy-atom bounds of Arg4, Phe5, Val27, Lys28, Tyr30, Tyr105, Met108 and Ala109, plus 5 Å on each face and the same minimum dimension. Exact production coordinates are listed in Table S4 and search_box_design.csv. These remain predicted surface-groove regions, not VOC co-crystal-defined pockets.

Validation used independent ETKDGv3 ligand coordinates rather than the native bound coordinates as the docking start. RMSD used RDKit CalcRMS in the fixed receptor frame with chemically valid automorphisms and heavy atoms only. The protocol, native-reference manifest, control logs and tabulated pose RMSDs were supplied. The subsequently supplied docking_v1 archive contains these native references, independently generated input coordinates, prepared files and raw pose outputs. Nine native/prepared control records matched their recorded hashes. All 200 tabulated retained validation-pose scores matched the raw Vina score remarks. The symmetry-aware RMSDs were not independently recomputed in this audit.

Supplementary Methods S4 Historical reconstruction and limits

Phase 4C verified normalization!E3 = E2/129, with the denominator labelled 129-mg. Workbook mass division is separate from the published CAP row/sample-sum standardization. November CAP outputs state no standardisation and 70 observations. The source supplement reports 63 biological observations, means of three technical chromatograms, 81 variables and 15 groups. The specimen crosswalk and reasons for exclusion remain unavailable.

The December sheet titled 91 bio contains 92 populated feature rows: 91 labels in column B plus Bio4 in A5. Eight rows were omitted and ten retained rows were edited, leaving 84 features. The available 75 × 84 consolidation and the separate Phase 5A.1 CAP workbook containing 76 × 84 values are historical stages. Neither is treated as the published 63 × 81 matrix. November row rearrangements were traced by RT/intensity vectors and annotation evidence rather than by same-row comparisons.

In the December all-lemon comparison, five measurement vectors moved from A18:F22 to A17:F21 while annotation cells G17 and G19 stayed in place. The former RT 33.149 row was absent. G17 therefore became associated with RT 35.432 while retaining beta farnesen. This spreadsheet-layout misalignment is independent of the later global-column-I import defect. Additional lemon-to-mint and DVBP-to-charcoal numerical transfers were reproducible, but their experimental purpose was not established.

The formula normalized-VOC!IT14 = IT13/45.5 references the previous normalized sample and yields 9321.441642. Division of the matched current raw input, normalization!IU26 = 32,220,308, by 45.5 gives 708,138.637363. The erroneous value occurs in both historical normalized matrices. Its use in the published

CAP analysis was not demonstrated. These historical matrices are not the numerical inputs to the revised presence-only V1 evidence layer.

Foxtail is labelled Alsail in the published Table S1 but Haw in Table S2. Feed is labelled Alkhalidiyah in S1 but Haw in S2. The conflicting source labels remain preserved. The Alhada rose taxonomic assignment also remains unresolved. No specimen-level barcode join is inferred. Multiplication by 51.13 and 29.62 is numerically reproducible, but the meanings of with IS, without IS and x factor, the internal-standard identity and amount, and the weighed material remain undocumented.

The complete chemical layer preserves the original published annotations and confidence symbols. Supplied validation tables report RDKit 2026.03.1 parsing and identifier consistency for all 55 resolved records. This audit independently checked the frozen-table joins and counts. It did not rerun RDKit or verify experimental identities. The 15 unresolved named candidates and 11 unknown features remain unassigned.

Raw-file consistency verification

An accompanying execution-level reproducibility archive (VOCBIO_V1_Docking_Reproducibility_FINAL.zip) is supplied within VOCBIO_Archive_Reconciled_Complete_Package.Zip as supplementary material. It contains receptor and ligand preparation assets, 200 production docking configurations, 200 raw AutoDock Vina outputs, 200 run logs and 2000 retained pose files. It also includes execution assets for the three control systems, frozen result tables, correction records, provenance manifests and file-level checksums. This archive complements the versioned VOC·BIO Library v1.0.0 software release at Zenodo (DOI: 10.5281/zenodo.23170512). Original software is MIT-licensed. Reuse of source-derived and third-party scientific material is governed separately by DATA_LICENSE.md and applicable source terms. The software and execution archives have distinct purposes. The frozen ZIP has SHA-256 a3eaf10ec88dcf2ca8385da58bd3e6b7ded2786f3b505486fb6f0a3e42c4e9aa. Its CHECKSUMS.sha256 file was independently checked against every listed packaged file, with no mismatches. Its MANIFEST.csv records 2806 payload files with matching authoritative expected hashes. Two recovered development-variant RMSD tables have packaged checksums but no authoritative expected hash. They remain explicitly marked RECOVERED_NO_AUTHORITATIVE_EXPECTED_HASH. The integrity audit does not constitute a new docking or RMSD calculation. Separately named aligned structures are not claimed as present.

Supplementary Methods S5 Platform evidence queries and computation

The unfiltered data layer comprises 81 unique published features, 15 unique contexts, 352 presence assertions, 70 chemical interpretations and 55 structure-enabled candidates. Features are joined to mappings one-to-one, and occurrence records join feature and context tables many-to-one. Context filtering returns a set of feature identifiers and then unique feature rows. Dashboard totals and confidence composition are calculated from those rows. Published GC-MS Features adds literal annotation and Bio search, numerical Bio ordering, context filters and CSV export. These operations preserve the distinction between feature count and presence-record count.

Context summaries use unique context identifiers and documentary species-host metadata. The exact host/material strings define the network host nodes. They are not a separately normalized botanical taxonomy. Historical sample masses remain a separate observation type. Their material definition, analytical roles and ambiguous workbook labels are not inferred.

Chemical Structure Explorer calculates 10 descriptors from eligible SMILES with RDKit 2026.03.1. The Molecular Weight display uses Descriptors.ExactMolWt, which is exact molecular mass rather than average molecular weight. Other descriptors are Wildman-Crippen LogP, TPSA, donor and acceptor counts, rotatable bonds, ring counts, aromatic rings, heavy atoms and fraction of sp3 carbon. The displayed radar axes use ceilings of 700, 12, 140, 10 and 25 for exact mass, LogP, TPSA, acceptors and rotatable bonds. Each plotted value is clipped to the interval zero to one after division. These display scales do not define a biological threshold. Structure pictures use PubChem image URLs; calculations use the locally stored candidate SMILES.

The structural similarity service generates Morgan fingerprints of radius 2 with 2048 bits. Tanimoto values are rounded to four decimal places. The threshold slider ranges from zero to one, with zero as its default. Invalid or out-of-bounds SMILES cannot be used for a calculation. Candidate confidence filters do not change the source labels. Similarity matrices and query results describe molecular fingerprints, not odour or function.

Published Feature Statistics deduplicates feature-context pairs before pivoting. The matrix is reindexed to all 81 feature IDs and 15 context IDs. Reported presence is one; unlisted presence is zero. Feature prevalence is row sum divided by 15. Confidence composition uses the inherited category. Jaccard context similarity is intersection divided by union, with a value of one for an empty union. The clustering service uses average linkage on the condensed distance matrix 1 - Jaccard. No biological hypothesis test, abundance transformation or CAP reproduction is performed.

Evidence Explorer returns source feature, occurrence, mapping and chemical records together. The feature-context network allows only context_species, context_host, published_presence and chemical_interpretation edges. The undirected graph uses a spring layout with seed 42 and k = 1.5 / sqrt(number of nodes). Displayed marker size is 10 + 2 × min(degree, 8). Node sizes and component counts reflect documentation connectivity. Filtered graph tables retain source and conflict fields. There are no attraction, repellency or binding edge types.

References / Data Sources describes the primary article and source Tables S1-S2. The assistant uses predefined keyword and Bio-ID branches and returns an answer, evidence layer, source, caution and record table. User text is limited to 300 characters, and custom chemical query input is limited to 512 characters before parsing. The assistant is deterministic and does not use an external LLM. A docking template in the archived v1.0.0 source still says that new docking results are unavailable. This is inconsistent with the frozen docking route and requires a versioned correction. Docking tables are the authoritative source of those reported calculations.

The frozen Docking Predictions explorer has eight subviews: overview, receptor-candidate matrix, candidate explorer, receptor comparison, seed variability, poses, validation, and methods/provenance. It reads offline outputs. It does not execute production docking or infer missing affinities. Preparation, docking, control and stability details remain in Supplementary Methods S2-S3 and Tables S2-S9.

Supplementary Methods S6 Independent platform consistency checks

The presence matrix was independently rebuilt from the frozen observation CSV using unique feature-context pairs. It exactly matched the archived statistics service. Feature and context keys were unique, the feature-chemical mapping was unique by feature, and no dangling feature/context references were found in the checked occurrence rows. The regenerated full-context network contains 190 nodes and 452 edges. Results are saved with the corresponding tables and platform_checks.json. These checks evaluate data consistency. Supplied structure-validation results are retained as supplied; local RDKit descriptor and fingerprint calculations were not rerun in this document-review environment. The software requirements and recorded scientific preparation environment remain separate.

Supplementary tables

Table S1 Published 81-feature evidence layer

Bio	Published RT	Published annotation	Published confidence notation
Bio4	10:26	α-Thujene ?	?
Bio5	10:45	Cyclofenchene ?	?
Bio10	12:24	β-Thujene ?	?
Bio19	14:77	O-Cymene ?	?

Bio	Published RT	Published annotation	Published confidence notation
Bio20	14:96	D-Limonene*	*
Bio21	15:08	Eucalyptol (1,8-cineole) *	*
Bio22	15:87	P-Cymene ?	?
Bio33	19:45	1,3,8-P-Menthatriene ?	?
Bio36	21:43	L-menthone ?	?
Bio37	21:78	D-menthone ?	?
Bio52	33:72	Caryophylene ?	?
Bio53	34:47	α-Guaiene ?	?
Bio55	35:30	Humulene ?	?
Bio56	35:46	(E)-β-Farnesene*	*
Bio59	36:75	β-Selinene ?	?
Bio61	37:14	trans-α-Bergamotene ?	?
Bio62	37:41	α-Bulnesene ?	?
Bio3	10:09	2-Methyl-4-heptanone ?	?
Bio13	13:07	6-Methyl-5-hepten-2-one ?	?
Bio57	35:65	2,6-Di-tert-butyl-p-benzoquinone ?	?
Bio8	11:91	Benzaldehyde*	*
Bio17	14:03	α-Methyl-benzeneacetaldehyde*	*
Bio31	18:95	Nonanal*	*
Bio2	9:63	1,1-Diacetoxyethane ?	?
Bio39	23:25	Methyl salicylate*	*
Bio66	41:32	2,2,4-Trimethylpentane-1,3-diyl bis(2-methylpropanoate) (TXIB)?? [wrapped label, PDF p5]	??
Bio77	55:60	Methyl pentacosanoate ??	??
Bio78	55:89	Methyl hexadecanoate ??	??
Bio81	58:84	Isopropyl palmitate ?	?
Bio15	13:64	Decane ?	?
Bio30	18:62	4,5-Dimethyl-nonane ??	??
Bio40	23:69	Dodecane ??	??
Bio43	26:88	1-Chloro-decane ?	?
Bio44	27:26	4,6-Dimethyl-dodecane ?	?
Bio45	28:55	Tridecane ??	??

Bio	Published RT	Published annotation	Published confidence notation
Bio47	29:41	Pentadecane*	*
Bio48	29:52	4,7-Dimethyl-undecane ??	??
Bio50	33:15	Tetradecane ??	??
Bio60	36:97	3,8-Dimethyl-undecane ??	??
Bio63	37:58	Hexadecane ?	?
Bio65	38:98	Nonadecane ?	?
Bio68	41:95	2,6,10,15-Tetramethylheptadecane ??	??
Bio71	46:27	Heptacosane ?	?
Bio72	48:03	Tetracosane ?	?
Bio74	50:35	Docosane ??	??
Bio75	50:48	Octacosane ??	??
Bio80	58:15	Tetratetracontane ?	?
Bio67	41:50	8-Heptadecene ?	?
Bio73	50:05	E-9-Eicosene ?	?
Bio1	8:80	β-methyl-Benzeneethanamine ??	??
Bio38	22:59	Azulene*	*
Bio41	24:73	Benzothiazole ??	??
Bio42	26:71	Caprolactam*	*
Bio49	32:68	1-Tetradecanol ?	?
Bio51	33:67	trans-Raphasatin ?	?
Bio54	34:69	cis-Raphasatin ?	?
Bio6	11:39	Bnz -6 ??	??
Bio7	11:72	Bnz -7 ??	??
Bio9	12:13	Mesitylene*	*
Bio11	12:49	Bnz -11 ??	??
Bio14	13:26	Bnz -14 ??	??
Bio16	13:90	Bnz-16 ??	??
Bio18	14:55	Bnz-18 ??	??
Bio23	16:04	Bnz -23 ??	??
Bio24	16:37	Bnz -24 ??	??
Bio26	16:80	Bnz -26 ??	??
Bio27	17:24	Bnz -27 ??	??

Bio	Published RT	Published annotation	Published confidence notation
Bio28	17:38	Bnz -28 ??	??
Bio29	17:69	Bnz -29 ??	??
Bio32	19:28	Bnz -32 ??	??
Bio12	12:97	UK-12	UK
Bio25	16:54	UK-25	UK
Bio34	20:72	UK-34	UK
Bio35	21:15	UK-35	UK
Bio46	28:86	UK-46	UK
Bio58	36:34	UK-58	UK
Bio64	38:18	UK-64	UK
Bio69	44:70	UK-69	UK
Bio70	46:16	UK-70	UK
Bio76	54:52	UK-76	UK
Bio79	57:74	UK-79	UK

Table S2 Frozen production ligand panel

Bio	Docking candidate	Published notation	Tier	CID	MW
Bio8	Benzaldehyde	*	Tier A	240	106.124
Bio9	Mesitylene	*	Tier A	7947	120.195
Bio17	2-Phenylpropanal	*	Tier A	7146	134.178
Bio20	D-Limonene	*	Tier A	440917	136.238
Bio21	Eucalyptol (1,8-cineole)	*	Tier A	2758	154.253
Bio31	Nonanal	*	Tier A	31289	142.242
Bio38	Azulene	*	Tier A	9231	128.174
Bio39	Methyl salicylate	*	Tier A	4133	152.149
Bio42	Caprolactam	*	Tier A	7768	113.16
Bio47	Pentadecane	*	Tier A	12391	212.421
Bio56	(E)-β-Farnesene	*	Tier A	5281517	204.357
Bio4	α-Thujene	?	Tier B	17868	136.238
Bio13	6-Methyl-5-hepten-2-one	?	Tier B	9862	126.199

Bio	Docking candidate	Published notation	Tier	CID	MW
Bio22	P-Cymene	?	Tier B	7463	134.222
Bio36	L-menthone	?	Tier B	26447	154.253
Bio49	1-Tetradecanol	?	Tier B	8209	214.393
Bio51	trans-Raphasatin	?	Tier B	5368086	159.279
Bio53	α-Guaiene	?	Tier B	5317844	204.357
Bio55	Humulene	?	Tier B	5281520	204.357
Bio59	β-Selinene	?	Tier B	442393	204.357

Full canonical SMILES, InChIKeys, published annotations and stereochemistry fields are retained in Table_S2_complete.csv. The complete 55-candidate structure layer is supplied in Table S11 and Table_S11_complete_55_structure_table.csv. The full frozen layer preserves all 81 features, including unresolved and UK records.

Table S3 Receptor provenance and preparation

PDB / protein	Species	Resolution Å	Chain	Missing residues	Preparation
4Z39 / MvicOBP3	Megoura viciae	1.3	A	none	PDBFixer/OpenMM then Meeko
4Z45 / NribOBP3	Nasonovia ribisnigri	2.02	A	LYS119;SER120;THR121	PDBFixer/OpenMM then Meeko

The full table retains preparation versions and caveats. Neither production structure contains a co-crystallized VOC. The 4Z45 terminal correction is documented in the preparation note and the source audit.

Table S4 Production search boxes

PDB	Center Å	Dimensions Å	Rationale
4Z39	8.284, 6.721, 22.849	27.811 × 29.469 × 22.853	Surface-groove residues + 5 Å margin; no co-crystallized VOC
4Z45	21.128, -20.253, 1.603	22.254 × 27.693 × 26.435	Surface-groove residues + 5 Å margin; no co-crystallized VOC

The residue-level box definition and frozen protocol are included in the accompanying frozen reproducibility archive. Search regions are predicted surface grooves, not experimentally validated VOC pockets.

Table S5 Independent redocking control summary

System	Role	Rank 1	Best of 10	Median rank-1 RMSD Å	Median best RMSD Å
1DQE	DEVELOPMENT	0/5	1/5	5.2674	2.3165
2WC6	HELDOUT	1/5	5/5	3.595	1.3438
3N7H	HELDOUT	5/5	5/5	0.6453	0.6453

Success means symmetry-corrected heavy-atom RMSD ≤2 Å. Sampling and ranking are reported separately. The controls benchmark the workflow on other OBPs. They do not validate either production binding region. Raw control poses, RMSD records and validation execution configurations are included in the accompanying reproducibility archive. Separately named aligned structures are not claimed as supplied assets.

Table S6 Complete production pair summaries

PDB	Bio	Published notation	Tier	Median score	Sample SD	Range
4Z39	Bio13	?	Tier B	-4.1300	0.0150	0.0300
4Z39	Bio17	*	Tier A	-5.1110	0.0067	0.0160
4Z39	Bio20	*	Tier A	-5.2100	0.0129	0.0310
4Z39	Bio21	*	Tier A	-5.0620	0.0138	0.0320
4Z39	Bio22	?	Tier B	-5.3070	0.0150	0.0390
4Z39	Bio31	*	Tier A	-4.0680	0.0671	0.1630
4Z39	Bio36	?	Tier B	-5.1430	0.0043	0.0110
4Z39	Bio38	*	Tier A	-4.4180	0.0080	0.0200
4Z39	Bio39	*	Tier A	-4.7170	0.0145	0.0360
4Z39	Bio4	?	Tier B	-4.8610	0.0034	0.0090
4Z39	Bio42	*	Tier A	-2.6240	0.0209	0.0490
4Z39	Bio47	*	Tier A	-4.5240	0.0596	0.1500
4Z39	Bio49	?	Tier B	-4.6160	0.1272	0.3220
4Z39	Bio51	?	Tier B	-3.5530	0.0237	0.0600
4Z39	Bio53	?	Tier B	-4.7630	0.1198	0.2850
4Z39	Bio55	?	Tier B	-4.2430	0.0445	0.1100
4Z39	Bio56	*	Tier A	-5.5540	0.1435	0.3640
4Z39	Bio59	?	Tier B	-6.7390	0.0065	0.0170
4Z39	Bio8	*	Tier A	-4.2270	0.0052	0.0140
4Z39	Bio9	*	Tier A	-4.7070	0.0057	0.0140
4Z45	Bio13	?	Tier B	-4.4460	0.0785	0.1970
4Z45	Bio17	*	Tier A	-5.2050	0.0307	0.0740
4Z45	Bio20	*	Tier A	-5.2210	0.0145	0.0320
4Z45	Bio21	*	Tier A	-5.3650	0.0058	0.0140
4Z45	Bio22	?	Tier B	-5.4040	0.0116	0.0290
4Z45	Bio31	*	Tier A	-4.2260	0.1243	0.2980
4Z45	Bio36	?	Tier B	-5.3400	0.0143	0.0340

PDB	Bio	Published notation	Tier	Median score	Sample SD	Range
4Z45	Bio38	*	Tier A	-4.4290	0.0254	0.0610
4Z45	Bio39	*	Tier A	-5.2420	0.0034	0.0090
4Z45	Bio4	?	Tier B	-5.0160	0.0115	0.0270
4Z45	Bio42	*	Tier A	-3.0160	0.0410	0.0900
4Z45	Bio47	*	Tier A	-4.5010	0.1783	0.4170
4Z45	Bio49	?	Tier B	-4.4430	0.2035	0.5380
4Z45	Bio51	?	Tier B	-3.4760	0.0691	0.1900
4Z45	Bio53	?	Tier B	-4.5160	0.0425	0.1100
4Z45	Bio55	?	Tier B	-4.3430	0.1208	0.3030
4Z45	Bio56	*	Tier A	-5.2600	0.2242	0.4900
4Z45	Bio59	?	Tier B	-6.4610	0.0203	0.0510
4Z45	Bio8	*	Tier A	-4.5630	0.0690	0.1730
4Z45	Bio9	*	Tier A	-5.0660	0.0047	0.0130

Table S7 Post hoc stability diagnostics

PDB	Bio	Score SD	Contact Jaccard	Largest cluster	Flag
4Z39	Bio13	0.0150	0.867	5/5	STABLE
4Z39	Bio17	0.0067	1.000	5/5	STABLE
4Z39	Bio20	0.0129	1.000	5/5	STABLE
4Z39	Bio21	0.0138	0.950	5/5	STABLE
4Z39	Bio22	0.0150	1.000	5/5	STABLE
4Z39	Bio31	0.0671	0.677	4/5	STABLE
4Z39	Bio36	0.0043	1.000	5/5	STABLE
4Z39	Bio38	0.0080	1.000	5/5	STABLE
4Z39	Bio39	0.0145	0.925	5/5	STABLE
4Z39	Bio4	0.0034	1.000	5/5	STABLE
4Z39	Bio42	0.0209	0.431	4/5	VARIABLE
4Z39	Bio47	0.0596	0.854	5/5	STABLE
4Z39	Bio49	0.1272	0.796	5/5	STABLE
4Z39	Bio51	0.0237	0.914	5/5	STABLE

PDB	Bio	Score SD	Contact Jaccard	Largest cluster	Flag
4Z39	Bio53	0.1198	0.757	5/5	STABLE
4Z39	Bio55	0.0445	0.964	5/5	STABLE
4Z39	Bio56	0.1435	0.919	5/5	STABLE
4Z39	Bio59	0.0065	1.000	5/5	STABLE
4Z39	Bio8	0.0052	0.950	5/5	STABLE
4Z39	Bio9	0.0057	0.956	5/5	STABLE
4Z45	Bio13	0.0785	0.789	5/5	STABLE
4Z45	Bio17	0.0307	0.700	5/5	STABLE
4Z45	Bio20	0.0145	1.000	5/5	STABLE
4Z45	Bio21	0.0058	1.000	5/5	STABLE
4Z45	Bio22	0.0116	1.000	5/5	STABLE
4Z45	Bio31	0.1243	0.816	5/5	STABLE
4Z45	Bio36	0.0143	0.945	5/5	STABLE
4Z45	Bio38	0.0254	0.840	5/5	STABLE
4Z45	Bio39	0.0034	1.000	5/5	STABLE
4Z45	Bio4	0.0115	0.775	5/5	STABLE
4Z45	Bio42	0.0410	0.765	5/5	STABLE
4Z45	Bio47	0.1783	0.727	5/5	STABLE
4Z45	Bio49	0.2035	0.770	5/5	VARIABLE
4Z45	Bio51	0.0691	0.796	5/5	STABLE
4Z45	Bio53	0.0425	0.867	5/5	STABLE
4Z45	Bio55	0.1208	0.880	5/5	STABLE
4Z45	Bio56	0.2242	0.874	5/5	VARIABLE
4Z45	Bio59	0.0203	1.000	5/5	STABLE
4Z45	Bio8	0.0690	0.721	5/5	STABLE
4Z45	Bio9	0.0047	1.000	5/5	STABLE

Table S8 Corrected representative contacts

PDB / Bio	Seed	Residue	Protein atom	Ligand atom	Distance Å
4Z39 / Bio56	104,729	MET3	O	C	3.398
4Z39 / Bio56	104,729	ARG4	CG	C	3.76
4Z39 / Bio56	104,729	PHE5	O	C	3.401
4Z39 / Bio56	104,729	ILE10	CD1	C	3.827

PDB / Bio	Seed	Residue	Protein atom	Ligand atom	Distance Å
4Z39 / Bio56	104,729	VAL27	O	C	3.357
4Z39 / Bio56	104,729	LYS28	O	C	3.323
4Z39 / Bio56	104,729	TYR30	C	C	3.651
4Z39 / Bio56	104,729	LYS31	O	C	3.61
4Z39 / Bio56	104,729	VAL32	CG2	C	3.898
4Z39 / Bio56	104,729	MET42	CE	C	3.927
4Z39 / Bio56	104,729	TYR105	CE1	C	3.593
4Z39 / Bio56	104,729	MET108	CE	C	4.197
4Z39 / Bio59	271,828	SER2	OG	C	3.428
4Z39 / Bio59	271,828	MET3	O	C	4.273
4Z39 / Bio59	271,828	ARG4	CA	C	3.504
4Z39 / Bio59	271,828	PHE5	O	C	3.356
4Z39 / Bio59	271,828	ILE10	CD1	C	3.591
4Z39 / Bio59	271,828	VAL27	CG1	C	3.52
4Z39 / Bio59	271,828	LYS28	O	C	3.267
4Z39 / Bio59	271,828	TYR30	OH	C	3.345
4Z39 / Bio59	271,828	MET42	CE	C	3.982
4Z39 / Bio38	314,159	ARG4	CG	C	3.664
4Z39 / Bio38	314,159	PHE5	O	C	3.536
4Z39 / Bio38	314,159	ILE10	CD1	C	3.746
4Z39 / Bio38	314,159	VAL27	CG1	C	3.626
4Z39 / Bio38	314,159	LYS28	CA	C	3.763
4Z39 / Bio38	314,159	TYR30	OH	C	3.7
4Z45 / Bio21	271,828	MET3	C	C	3.78
4Z45 / Bio21	271,828	ARG4	CG	O	3.457
4Z45 / Bio21	271,828	PHE5	N	C	3.758
4Z45 / Bio21	271,828	TYR30	CD1	C	3.57
4Z45 / Bio21	271,828	MET42	CE	C	3.665
4Z45 / Bio21	271,828	TYR105	CD1	C	3.761
4Z45 / Bio21	271,828	MET108	CE	C	3.572
4Z45 / Bio21	271,828	ASN112	ND2	C	3.597
4Z45 / Bio39	104,729	SER2	OG	O	2.992

PDB / Bio	Seed	Residue	Protein atom	Ligand atom	Distance Å
4Z45 / Bio39	104,729	MET3	N	O	3.176
4Z45 / Bio39	104,729	ARG4	N	O	2.905
4Z45 / Bio39	104,729	TYR30	CB	O	3.591
4Z45 / Bio39	104,729	MET42	CE	C	3.382
4Z45 / Bio39	104,729	TYR105	CE1	C	4.209
4Z45 / Bio39	104,729	MET108	CB	C	3.738
4Z45 / Bio39	104,729	ASN112	ND2	O	3.022
4Z45 / Bio38	314,159	MET3	O	C	3.674
4Z45 / Bio38	314,159	ARG4	CA	C	3.579
4Z45 / Bio38	314,159	PHE5	N	C	3.499
4Z45 / Bio38	314,159	TYR30	CD1	C	3.63
4Z45 / Bio38	314,159	MET42	CE	C	3.646
4Z45 / Bio38	314,159	TYR105	CD1	C	3.779
4Z45 / Bio38	314,159	MET108	CE	C	3.79

Full interaction types, geometric thresholds and caveats are in corrected_representative_interactions.csv. These rows record predicted-complex proximity, not measured interactions. The corrected NribOBP3-Bio39 distances are 2.905 Å for ARG4 N and 3.591 Å for TYR30 CB to ligand oxygen.

Table S9 Source stereochemistry caveats

Bio	Candidate	Source specifies stereochemistry	Interpretation
Bio17	2-Phenylpropanal	no	One coordinate realization; no experimental stereoisomer assigned
Bio4	α-Thujene	no	One coordinate realization; no experimental stereoisomer assigned
Bio21	Eucalyptol (1,8-cineole)	no	One coordinate realization; no experimental stereoisomer assigned

Table S10 Published sample-context metadata

Species	Accession	Host / material	Site	Date
Aphis punicae	MZ091379	Leaves and stems of pomegranates	Green house (Alhawia)	27-6-2021
Aphis gossypii	MZ091376	Leaves of zucchini	Green house (Alhawia)	21-11-2020
Aphis gossypii	MZ091376	Leaves of vinca	Alhawia	21-11-2020
Aphis gossypii	MZ091376	Flower buds of hibiscus plant	Alhawia	21-11-2020
Aphis gossypii	MZ091376	Leaves of lemon	Alkhalidiyah	21-5-2021

Species	Accession	Host / material	Site	Date
Aphis gossypii	MZ091376	mint	Alkhalidiyah	21-5-2021
Aphis gossypii	MZ091376	fresh Feed	Alkhalidiyah	21-5-2021
Aphis craccivora	MZ091375	Leaves of Alfalfa	Alhawia	22-6-2021
Rhodobium porosum	MZ091382	Leaves and stems of Taif rose	Green house (Alhawia)	22-6-2021
Macrosiphum rosae	OL823183	Leaves and stems of Taif rose	Alhada	11-9-2021
Aphis illinoisensis	MZ091377	Leaves and stems of grape	Alsir	21-5-2021
Aphis nerii	MZ091378	Flowers of oleander	Alhawia	19-6-2021
Eriosoma lanigerum	OL823181	Stems of green apple	Alhada	20-8-2021
Lipaphis pseudobrassicae	MZ091380	Leaves of radish	Alhawia	21-11-2020
Hysteroneura setariae	OL823182	Flower of green foxtail	Alsail	11-7-2021

Source: Supplementary Table S1 of Alotaibi et al. (2023). This reproduces published context metadata, not specimen-level barcode confirmation. Foxtail and feed locality labels conflict between the source Tables S1 and S2. Alhada rose workbook labels conflict with the molecular context assignment. Both conflicts remain documented in the audit.

Table S11 Complete frozen structure-enabled candidate list

Bio	Candidate	Published notation	Tier	CID
Bio1	2-Phenylpropan-1-amine	??	Tier C	11398
Bio2	1,1-Diacetoxyethane	?	Tier B	222536
Bio3	2-Methyl-4-heptanone	?	Tier B	69378
Bio4	α-Thujene	?	Tier B	17868
Bio5	Cyclofenchene	?	Tier B	79022
Bio8	Benzaldehyde	*	Tier A	240
Bio9	Mesitylene	*	Tier A	7947
Bio10	β-Thujene	?	Tier B	520384
Bio13	6-Methyl-5-hepten-2-one	?	Tier B	9862
Bio15	Decane	?	Tier B	15600
Bio17	2-Phenylpropanal	*	Tier A	7146
Bio19	O-Cymene	?	Tier B	10703
Bio20	D-Limonene	*	Tier A	440917
Bio21	Eucalyptol (1,8-cineole)	*	Tier A	2758
Bio22	P-Cymene	?	Tier B	7463
Bio30	4,5-Dimethylnonane	??	Tier C	86541

Bio	Candidate	Published notation	Tier	CID
Bio31	Nonanal	*	Tier A	31289
Bio33	1,3,8-P-Menthatriene	?	Tier B	176983
Bio36	L-menthone	?	Tier B	26447
Bio37	D-menthone	?	Tier B	443159
Bio38	Azulene	*	Tier A	9231
Bio39	Methyl salicylate	*	Tier A	4133
Bio40	Dodecane	??	Tier C	8182
Bio41	Benzothiazole	??	Tier C	7222
Bio42	Caprolactam	*	Tier A	7768
Bio43	1-Chloro-decane	?	Tier B	13848
Bio44	4,6-Dimethyldodecane	?	Tier B	545627
Bio45	Tridecane	??	Tier C	12388
Bio47	Pentadecane	*	Tier A	12391
Bio48	4,7-Dimethylundecane	??	Tier C	519389
Bio49	1-Tetradecanol	?	Tier B	8209
Bio50	Tetradecane	??	Tier C	12389
Bio51	trans-Raphasatin	?	Tier B	5368086
Bio53	α-Guaiene	?	Tier B	5317844
Bio54	(Z)-4-Isothiocyanato-1-methylsulfanylbut-1-ene	?	Tier B	91699052
Bio55	Humulene	?	Tier B	5281520
Bio56	(E)-β-Farnesene	*	Tier A	5281517
Bio57	2,6-Di-tert-butyl-p-benzoquinone	?	Tier B	12867
Bio59	β-Selinene	?	Tier B	442393
Bio60	3,8-Dimethylundecane	??	Tier C	86540
Bio61	trans-α-Bergamotene	?	Tier B	6429302
Bio62	α-Bulnesene	?	Tier B	94275
Bio63	Hexadecane	?	Tier B	11006
Bio65	Nonadecane	?	Tier B	12401
Bio66	2,2,4-Trimethylpentane-1,3-diyl bis(2-methylpropanoate) (TXIB)	??	Tier C	23284
Bio68	2,6,10,15-Tetramethylheptadecane	??	Tier C	41209
Bio71	Heptacosane	?	Tier B	11636
Bio72	Tetracosane	?	Tier B	12592

Bio	Candidate	Published notation	Tier	CID
Bio73	E-9-Eicosene	?	Tier B	5365037
Bio74	Docosane	??	Tier C	12405
Bio75	Octacosane	??	Tier C	12408
Bio77	Methyl pentacosanoate	??	Tier C	41431
Bio78	Methyl hexadecanoate	??	Tier C	8181
Bio80	Tetratetracontane	?	Tier B	23494
Bio81	Isopropyl palmitate	?	Tier B	8907

Complete SMILES, InChI, InChIKeys, formulas, molecular weights, source records, curation notes and uncertainty fields are supplied in Table_S11_complete_55_structure_table.csv. The unresolved records are retained in frozen_v1_chemical_layer.csv and remaining_unresolved_structures.csv. Structure availability does not confirm a tentative peak identification.

Table S12 Computational roles of all 11 platform features

Feature	Input	Calculation or retrieval
Dashboard	Linked evidence tables	Counts, confidence composition and reported prevalence
Published GC-MS Features	81 source features	Search, context/confidence filters and CSV export
Chemical Structure Explorer	55 candidate structures	Identifiers, depictions and 10 molecular descriptors
Biological & Sample Contexts	15 source contexts	Metadata filtering and species-host summaries
Published Feature Statistics	352 presence assertions	81 × 15 matrix, Jaccard similarity and clustering
Evidence Explorer	Feature-context-chemical joins	Source annotation, confidence and provenance packet
Docking Predictions	Frozen offline analysis	40 pairs, five seeds, scores, poses and validation
Feature-Context Network	Typed evidence links	Graph construction, degree and component summaries
Structural Similarity Explorer	Structure-enabled candidates	Morgan/Tanimoto searches and pairwise comparison
References / Data Sources	Primary article and supplement	Source-table roles and confidence definitions
Research Team Assistant	Supported record queries	Deterministic templates with sources and cautions

The main manuscript presents the same feature inventory in Table 1. Matrix and network counts refer to all contexts with all confidence categories included. The interface network defaults to a selected context, so its displayed totals can be smaller. All 26 features without resolved candidate structures remain in source-feature and context calculations.

Supplementary figures

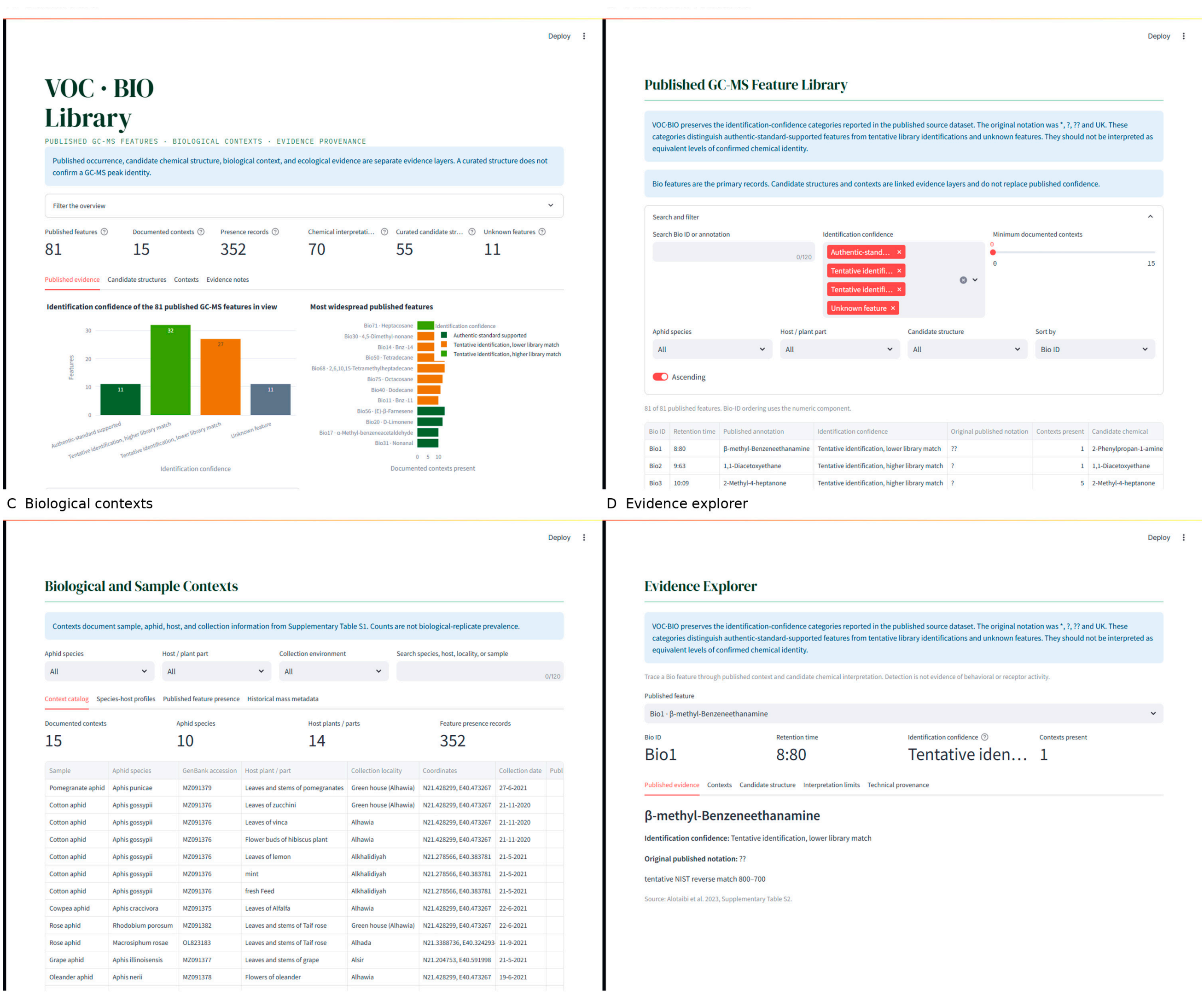

Figure S1. Core application views. Published features, contexts and provenance remain separate from candidate structures.

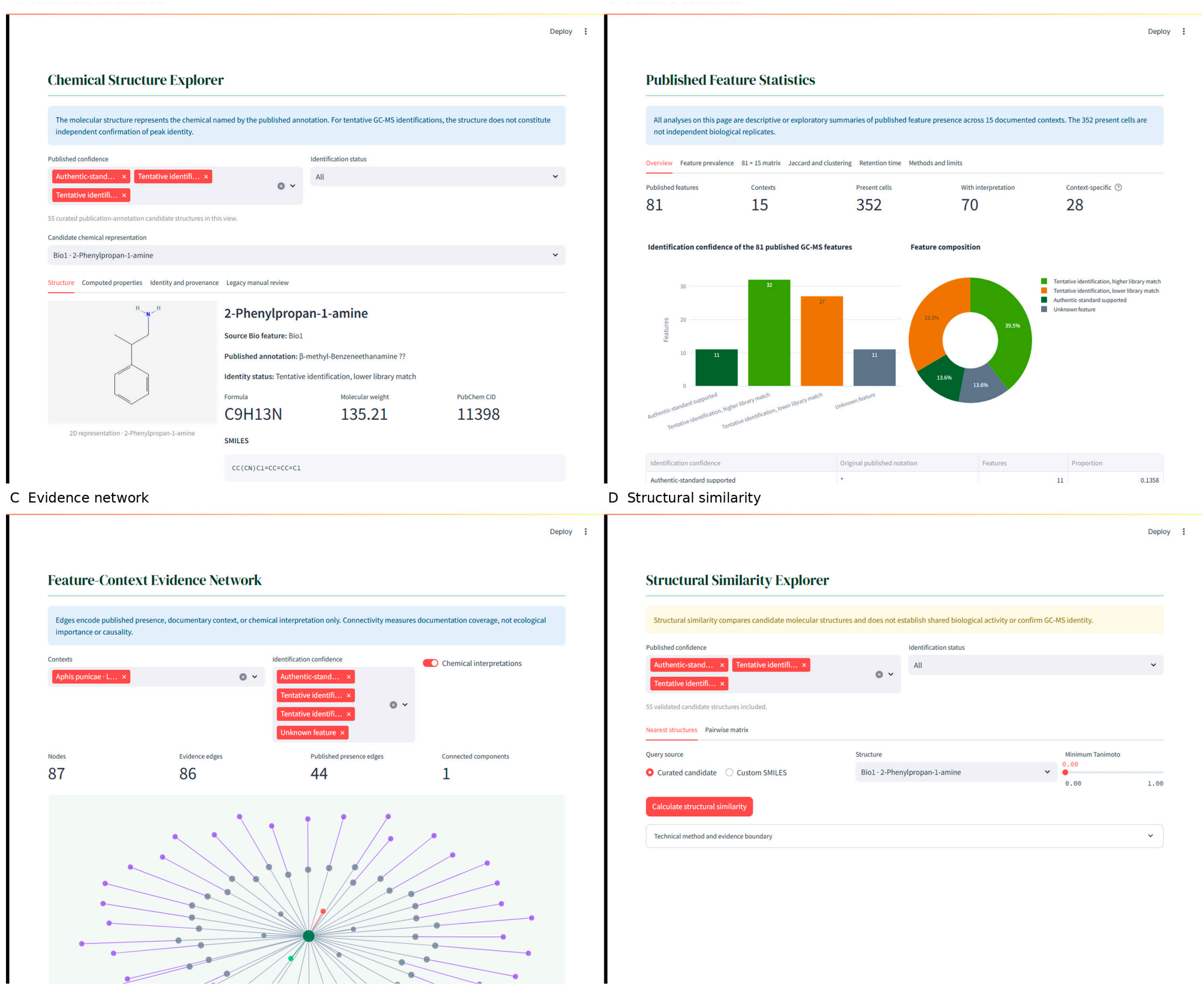

Figure S2. Evidence, cheminformatics and network views. These descriptive displays do not confirm chemical identity or ecological causality.

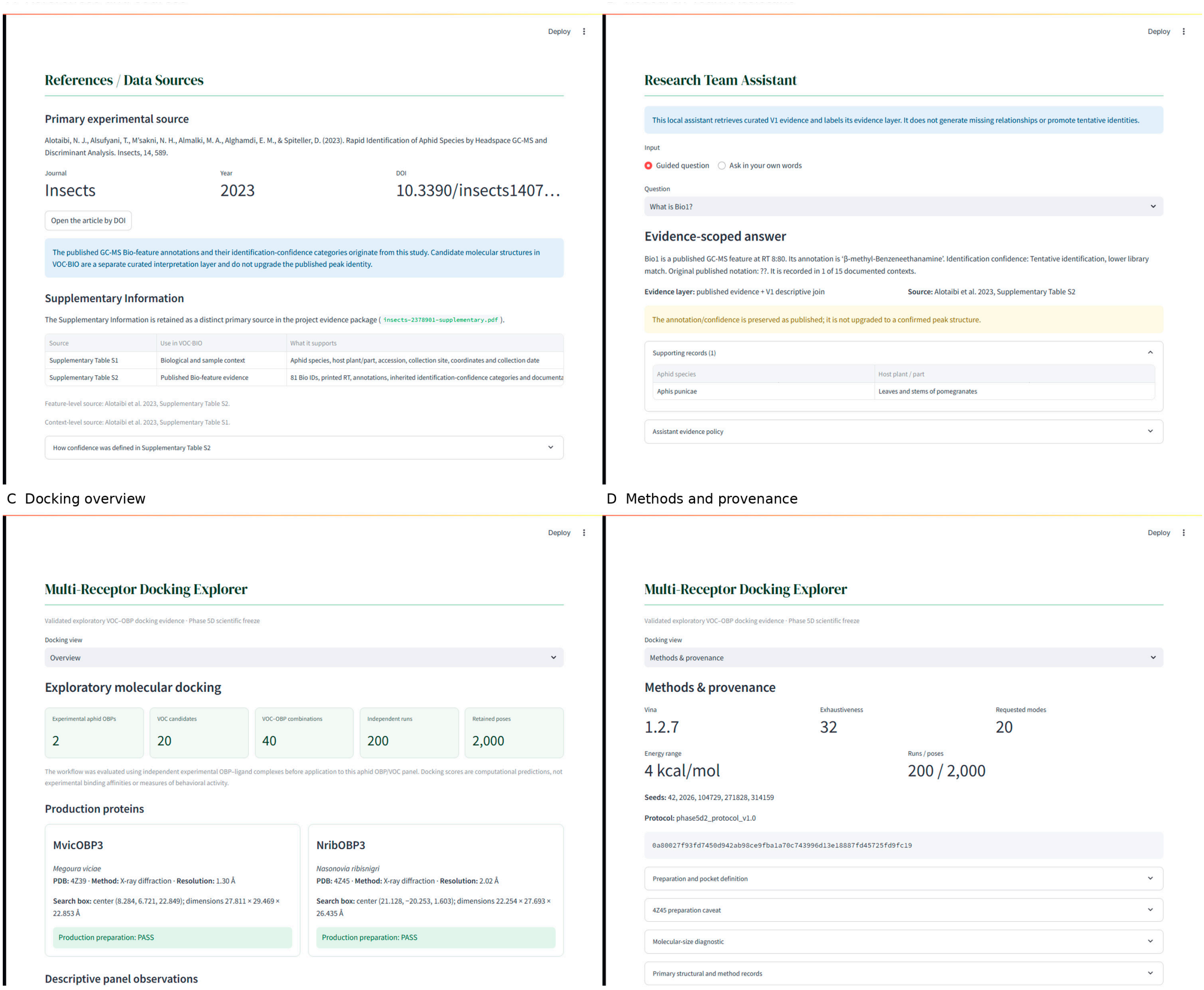

Figure S3. Reference, guided-query and docking-provenance views from the supplied Version 1.0 interface captures. The current docking route is separate from the assistant template limitation described in Supplementary Methods S5.

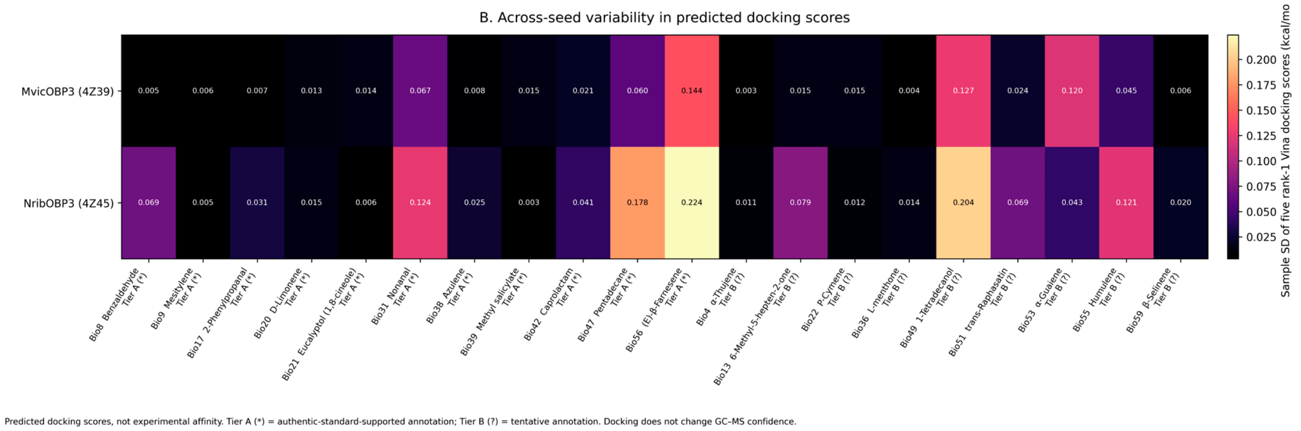

Figure S4. Sample SD of rank-1 scores across five seeds. Values are computational variability, not biological variance.

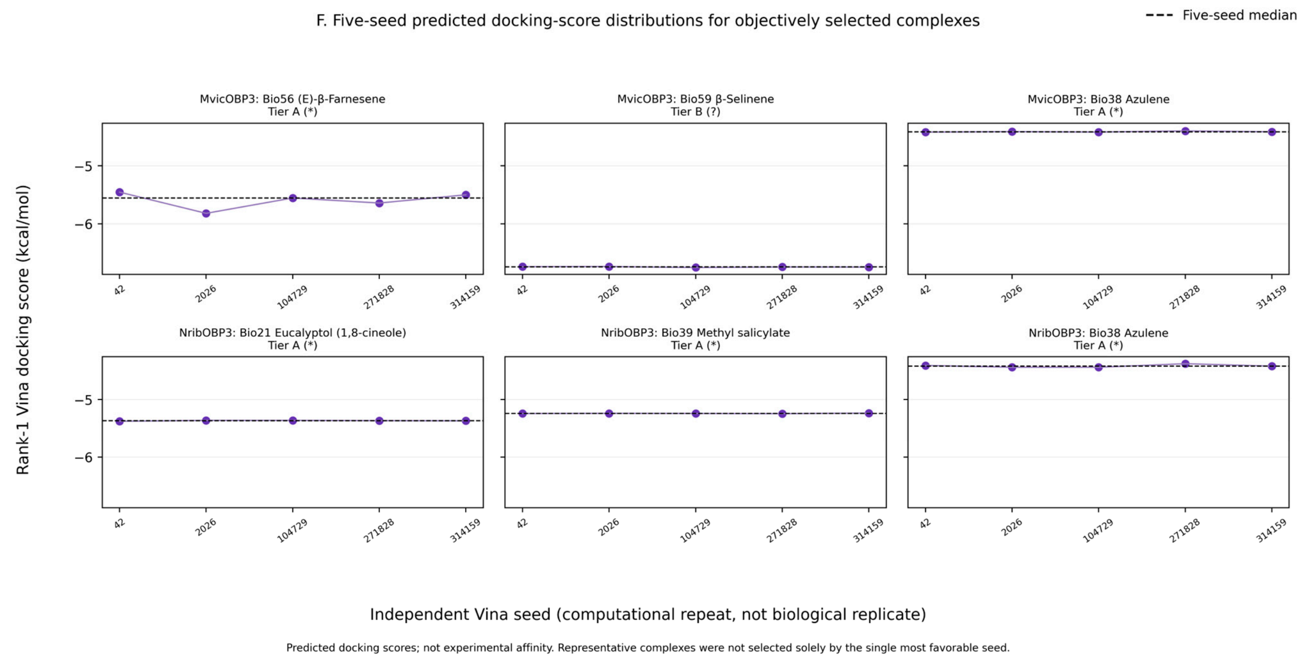

Figure S5. Selected five-seed score distributions. Seeds are computational repeats.

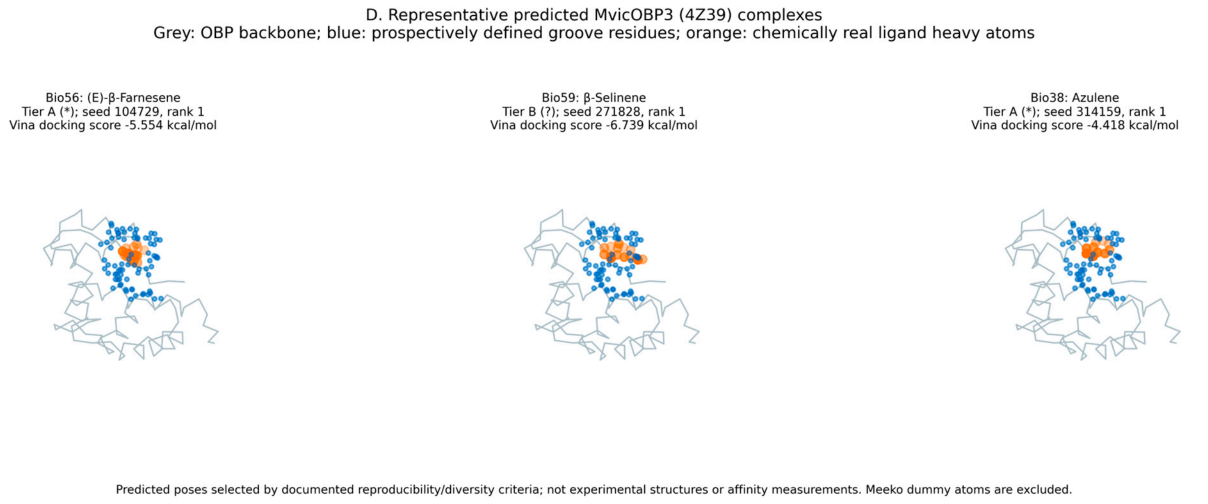

Figure S6. Representative MvicOBP3 poses selected by the documented rule. Dummy ring-closure atoms are excluded. Predicted geometry does not establish a binding mode.

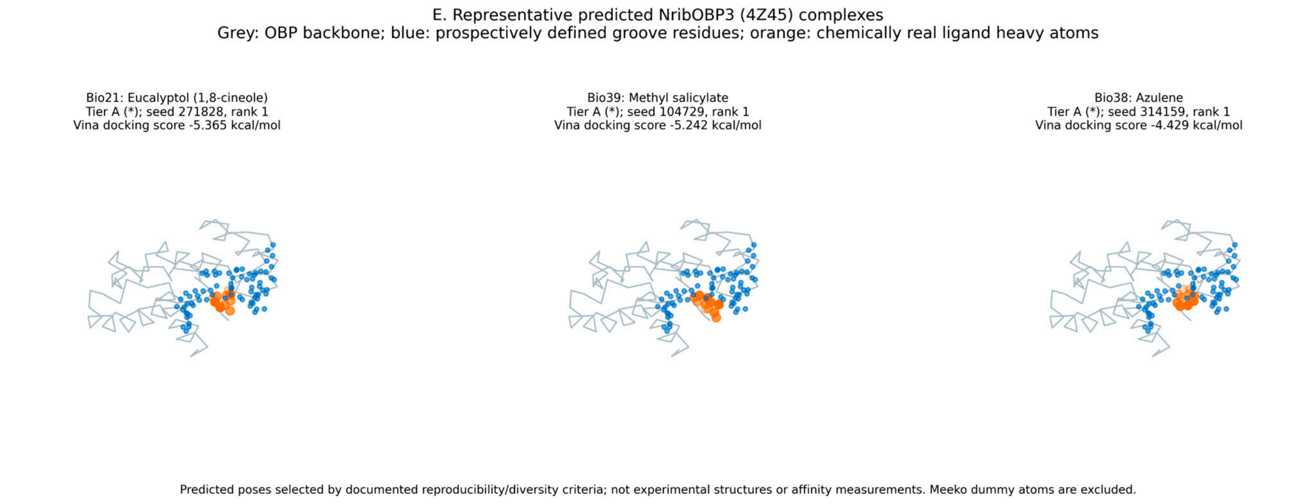

Figure S7. Representative NribOBP3 poses. The corrected heavy-atom contact annotations are in Table S8 and the full CSV.

Interpretation and reproducibility

Vina scores are not measured affinity, Ki, activation, inhibition, behavior, ecological function or pest-control efficacy. The selected OBPs are not from the ten source GC-MS species. Neither production OBP contains a co-crystallized VOC. Five seeds are computational repeats, and stability thresholds are post hoc diagnostics. The complete chemical table, frozen control protocol, production box definitions and validation summary records accompany this supplement. An accompanying execution-level reproducibility archive (VOCBIO_V1_Docking_Reproducibility_FINAL.zip) is supplied within VOCBIO_Archive_Reconciled_Complete_Package.Zip as supplementary material. It contains receptor and ligand preparation assets, 200 production docking configurations, 200 raw AutoDock Vina outputs, 200 run logs and 2000 retained pose files. It also includes execution assets for the three control systems, frozen result tables, correction records, provenance manifests and file-level checksums. This archive complements the versioned VOC·BIO Library v1.0.0 software release at Zenodo (DOI: 10.5281/zenodo.23170512). Original software is MIT-licensed. Reuse of source-derived and third-party scientific material is governed separately by DATA_LICENSE.md and applicable source terms. The software and execution archives have distinct purposes. The frozen ZIP has SHA-256 a3eaf10ec88dcf2ca8385da58bd3e6b7ded2786f3b505486fb6f0a3e42c4e9aa. Its CHECKSUMS.sha256 file was independently checked against every listed packaged file, with no mismatches. Its MANIFEST.csv records 2806 payload files with matching authoritative expected hashes. Two recovered development-variant RMSD tables have packaged checksums but no authoritative expected hash. They remain explicitly marked RECOVERED_NO_AUTHORITATIVE_EXPECTED_HASH. The integrity audit does not constitute a new docking or RMSD calculation. Separately named aligned structures are not claimed as present.

References

1. Alotaibi, N.J.; Alsufyani, T.; M’sakni, N.H.; Almalki, M.A.; Alghamdi, E.M.; Spiteller, D. Rapid identification of aphid species by headspace GC-MS and discriminant analysis. *Insects* **2023**, *14*, 589. https://doi.org/10.3390/insects14070589.
